# Supplementary material for: Intensity modulation of trichromatic split fluorescent proteins for live cell mapping
Source: Cell Rep Methods. 2026 Mar 26;6(4):101363. doi: 10.1016/j.crmeth.2026.101363 (PMC13107049; doi:10.1016/j.crmeth.2026.101363)
Supplement: Document S1. Figures S1–S5 and Table S1 [file mmc1.pdf]

Cell Reports Methods, Volume *6*

Supplemental information

Intensity modulation of trichromatic split fluorescent proteins for live cell mapping

Mamoru Ishii, Tomoaki Kinjo, Yohei Kondo, Kenta Terai, Kazuhiro Aoki, Brian Kuhlman, and Michiyuki Matsuda

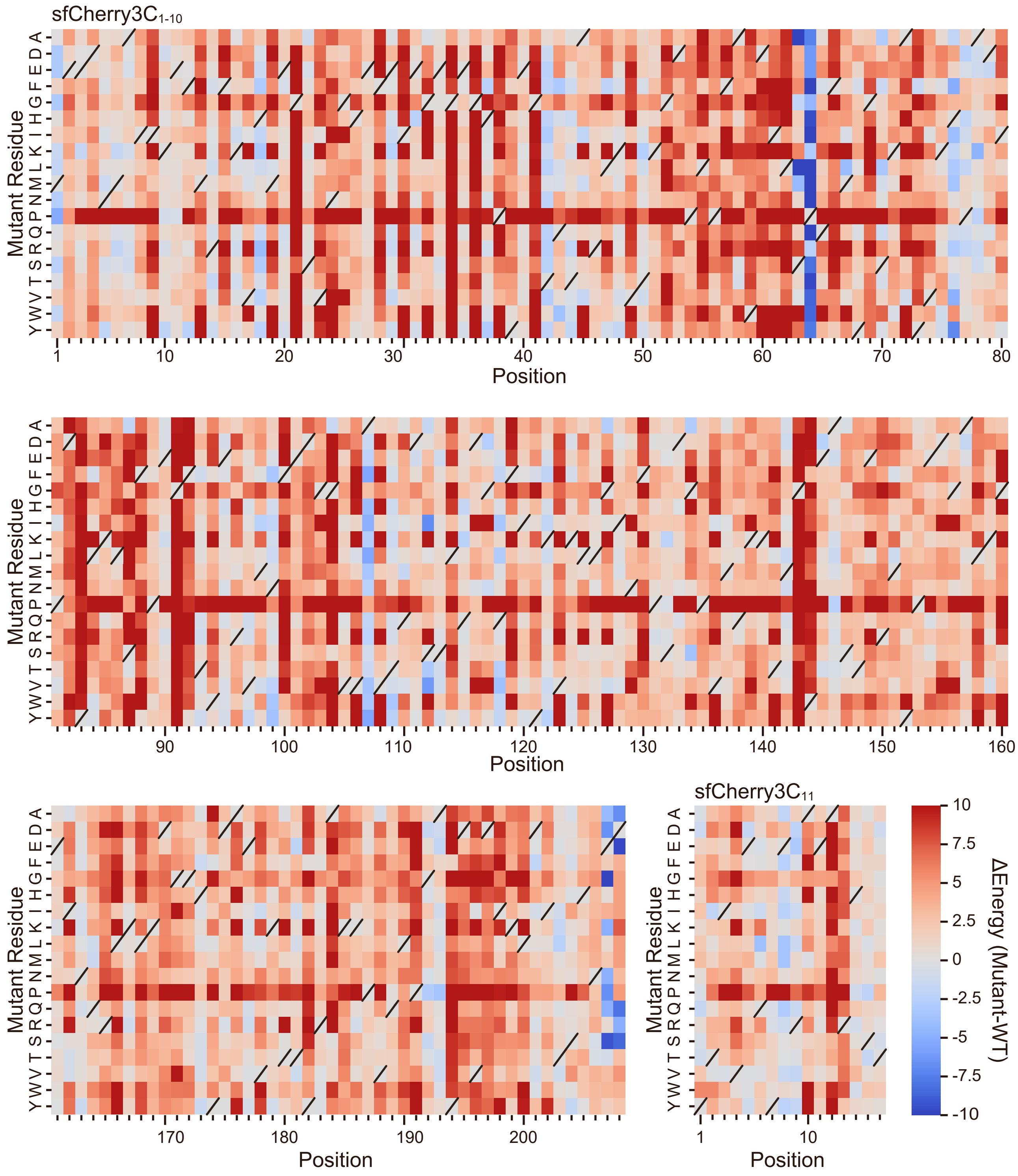

Figure S1: Comprehensive mutational scanning of split sfCherry3C, related Figure 3.

Heatmap displaying the ΔEnergy values for saturating mutations in both $\text{sfCherry}_{1\text{-}10}$ and sfCherry_{11}. Black slashes indicate wild-type amino acids.

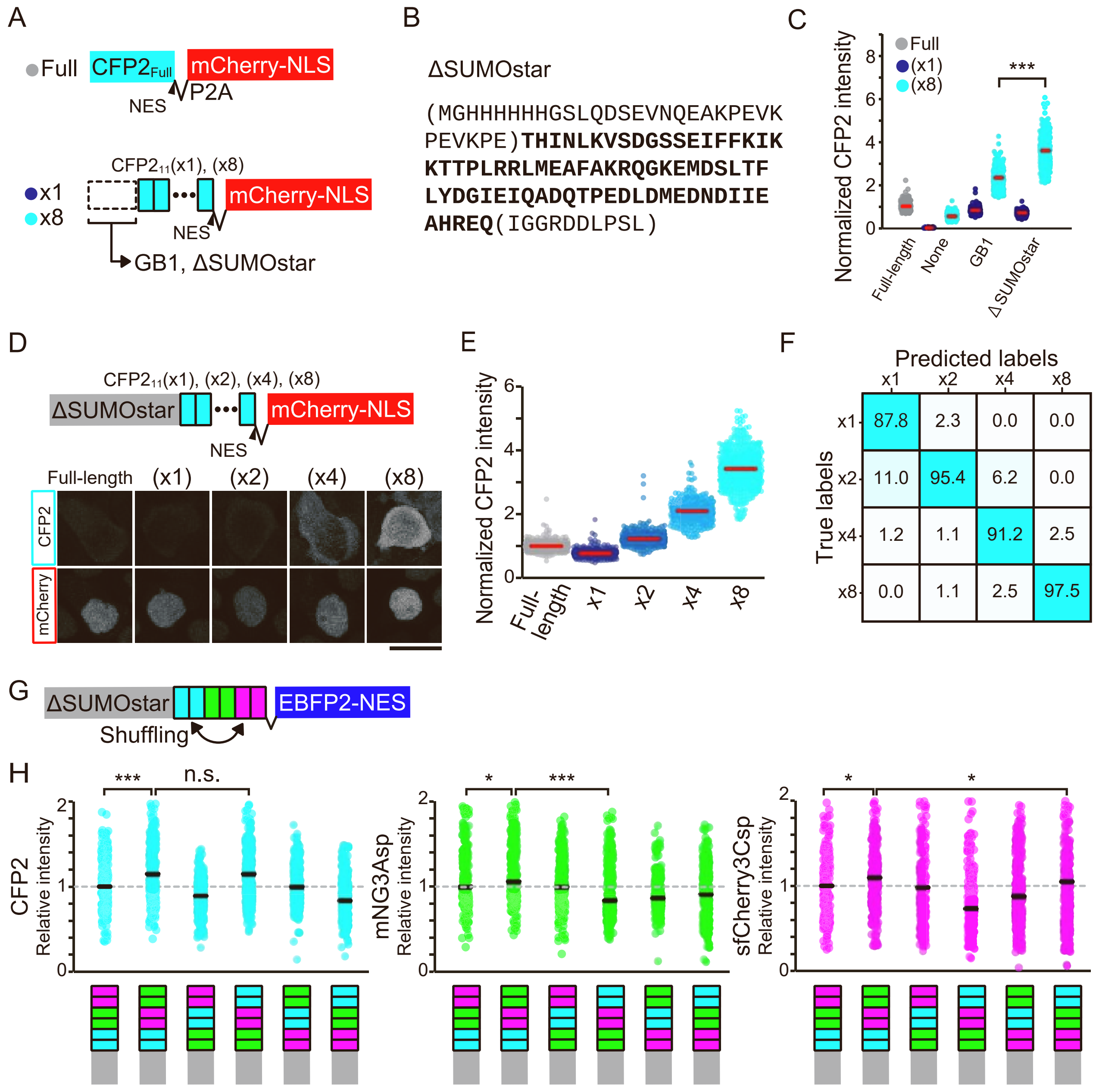

Figure S2: Optimization of the FP$_{11}$ tag, related to Figure 5.

(A) Schematic representation of expression constructs: CFP2$_{\text{Full-length}}$-NES and CFP2$_{11}$ variants [(x1) or (x8)]-NES with or without GB1 or ΔSUMOstar (truncated SUMOstar) tags.
(B) Amino acid sequences of SUMOstar, with ΔSUMOstar region highlighted in bold.
(C) Bee swarm plots showing quantitative analysis of normalized CFP2 fluorescence in HeLa cells co-expressing CFP2$_{1\text{-}10}$ with either CFP2$_{\text{Full-length}}$-NES or CFP2$_{11}$(x1)/CFP2$_{11}$(x8)-NES variants, with or without GB1 or ΔSUMOstar tags (constructs shown in panel A). Median values are indicated by red lines.
(D) Construct architecture and expression analysis: Upper panel shows schematics of ΔSUMOstar fusions containing varying copy numbers of CFP2$_{11}$ [CFP2$_{11}$(x1), (x2), (x4), and (x8)]. Lower panel presents representative confocal micrographs of HeLa cells stably co-expressing CFP2$_{1\text{-}10}$ with either CFP2$_{\text{Full-length}}$-NES or ΔSUMOstar-tagged CFP2$_{11}$ variant. Scale bar: 20 µm.
(E) Comparative analysis of normalized CFP2 fluorescence in HeLa cells expressing ΔSUMOstar-tagged CFP2$_{11}$ variants [(x1), (x2), (x4), or (x8)] shown in panel D. Data presented as bee swarm plots with median values indicated by red lines.
(F) Classification performance matrix showing predictive accuracy against true population identities (rows), with color intensity indicating classification accuracy.
(G) Schematic representation of expression constructs: ΔSUMO-tagged FP$_{11}$ arrays comprising paired repeats of CFP2$_{11}$, mNG3Asp$_{11}$, and sfCherry3Csp$_{11}$ [(x2) each, total (x6)].
(H) Quantitative analysis presented as bee swarm plots showing normalized fluorescence intensities (CFP2, mNG3Asp, and sfCherry3Csp) in HeLa cells expressing six different permutations of FP$_{11}$ repeat arrangements, with concurrent FP$_{1\text{-}10}$x3 expression. Data obtained by confocal microscopy, with median values indicated by black lines.

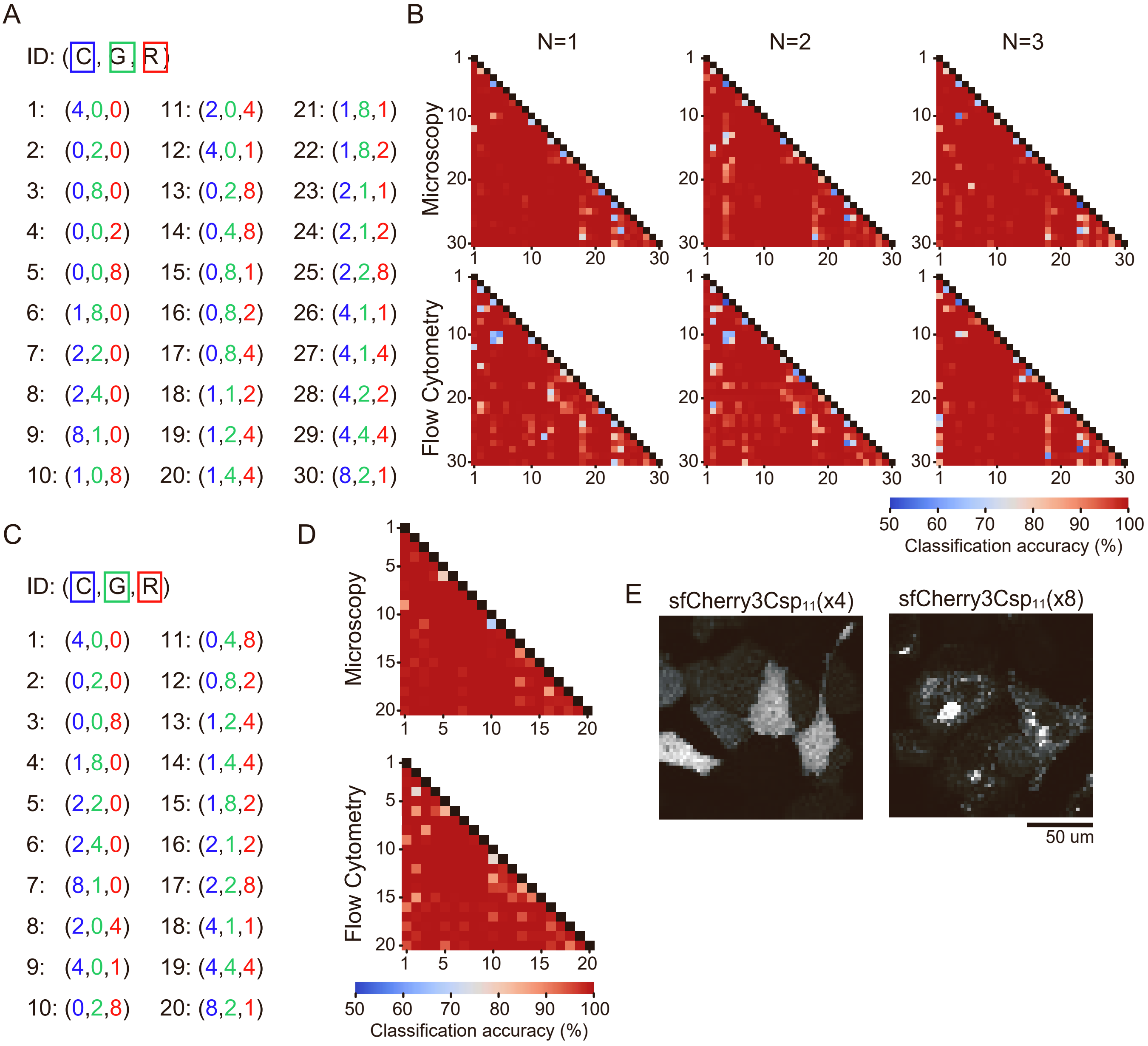

Figure S3: Selection of 20 optimal FP_{11} tags, related to Figure 5.

(A) Catalog of 30 selected FP_{11} tag variants (detailed in Figure 5A), detailing identification numbers and copy numbers of $CFP2_{11}$, $mNG3Asp_{11}$, and $sfCherry3Csp_{11}$ fragments.
(B) Matrix analysis of pairwise classification accuracies for 30 cell populations expressing distinct FP_{11} tag combinations (listed in panel A), evaluated by both microscopy (upper panel) and flow cytometry (lower panel). Data represent results from three independent experiments.
(C) Catalog of optimized FP_{11} tag set comprising 20 selected variants (detailed in Figure 5A), detailing identification numbers and copy numbers of $CFP2_{11}$, $mNG3Asp_{11}$, and $sfCherry3Csp_{11}$ fragments.
(D) Matrix analysis of pairwise classification accuracy for 20 cell populations expressing distinct FP_{11} tag combinations (defined in panel C). Comparative evaluation performed using both microscopy (upper panel) and flow cytometry (lower panel). Data represent mean values from three independent experiments in panel B.
(E) Representative images of sfCherry3Csp fluorescence in HeLa cells expressing ΔSUMOstar-tagged $sfCherry3Csp_{11}$ variants [(x4), or (x8)], with concurrent $FP_{1\text{-}10}x3$ expression.

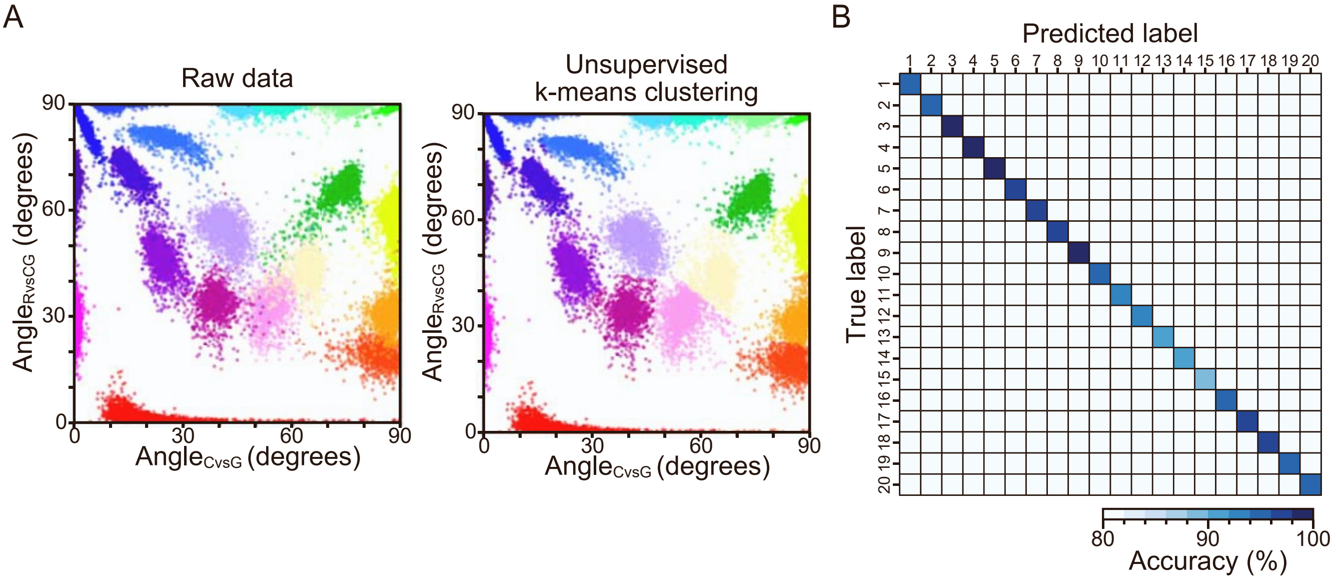

Figure S4: Unsupervised k-means clustering of 20 cell populations, related to Figure 6.

(A) Left: Scatter plot showing the distribution of Angle$_{CvsG}$ versus Angle$_{RvsCG}$ for 20 distinct cell populations (n > 1,000 cells per population). Right: Scatter plot showing the distribution of Angle$_{CvsG}$ versus Angle$_{RvsCG}$ for individual cells grouped into 20 distinct populations using unsupervised k-means clustering.

(B) Classification performance matrix of unsupervised k-means classification in panel A. Color intensity indicates classification accuracy. Overall average accuracy, 96 %.

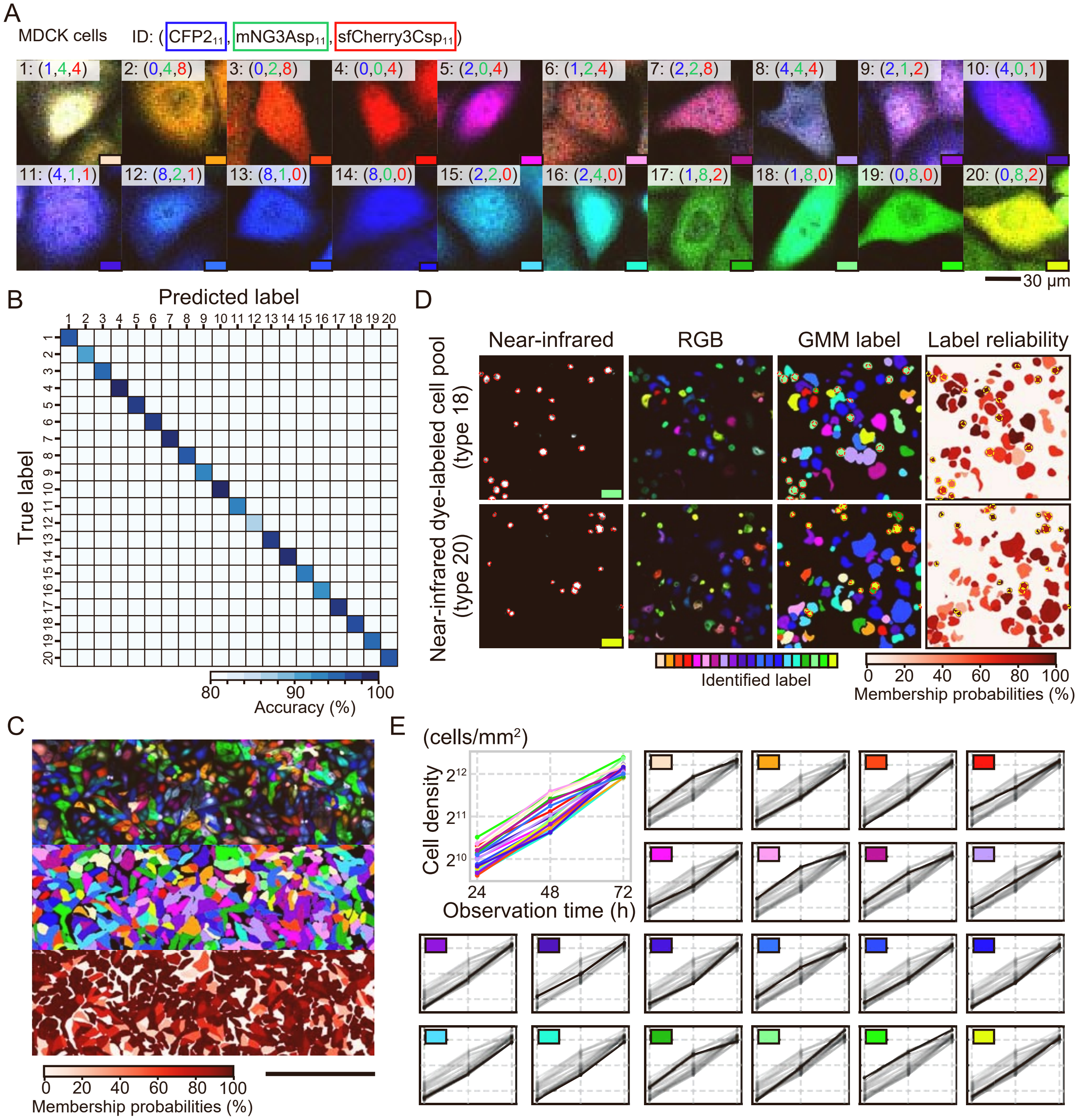

Figure S5: Accurate multicolor classification and normal growth of labeled MDCK cells, related to Figure 7.

(A) Representative multicolor fluorescence micrographs of 20 distinct cell populations expressing FP_{11} tags. Fluorescence channels: CFP2 (blue), mNG3Asp (green), and sfCherry3Csp (red). Copy numbers of each FP_{11} variant ($CFP2_{11}$, $mNG3Asp_{11}$, and $sfCherry3Csp_{11}$) are indicated in the top of each panel. Scale bars: 30 μm.
(B) Classification performance matrix showing prediction accuracy against true population identities (rows). Color intensity indicates classification accuracy. Overall average accuracy, 96%.
(C) Top: Large-field composite image of pooled cell populations from panel (A), displaying CFP2 (blue), mNG3Asp (green), and sfCherry3Csp (red) fluorescence channels. Scale bar: 500 μm. Middle: Population assignment map following GMM-based classification into 20 distinct populations. Bottom: Visualization of GMM classification confidence through membership probability mapping.
(D) From left to right: near-infrared dye–labeled cells stained with DRAQ5, RGB composite image of the pooled 20 cell populations, GMM label map, and GMM label reliability map. The upper panel shows type 18 cells (B,G,R = 1,8,0) and the lower panel shows type 20 cells (B,G,R = 0,8,2), both labeled with the near-infrared dye. Among 81 total cells (type 18, upper panel), the classifier identified 20 true positives, 1 false positive, 1 false negative, and 59 true negatives, yielding a sensitivity of 95.2% (20/21) and specificity of 98.3% (59/60). Among 93 total cells (type 20, lower panel), there were 17 true positives, 0 false positives, 2 false negative, and 75 true negatives, yielding a sensitivity of 89.5% (17/19) and specificity of 100% (75/75).
(E) Growth curves showing cell density of 20 different MDCK cell types fluorescently labeled with the Caterpie method measured every 24 hours. The each panel displays the individual growth curve.

Supplementary Table 1: ***Amino acid sequences of the $FP_{1\text{-}10}$ and FP_{11} tag, related to Figure 6.***

	**Amino acid sequences**
$CFP2_{1\text{-}10}$	MSKGEELFTGVVPILVELEGEVNGHKFSVRGEGEGDATIGKLTLKFICT TGKLPVPWPTLVTTLTWGVQCFSRYPDHMKRHDFFKSAMPEGYVQE RTISFKDDGKYKTRAVVKFEGDTLVNRIVLKGTDFKEDGNILGHKLEYN FNSDNVYITADKQKNGIKANFTIRHNVEDGSVQLADHYQQNTPIGDGP VLLPDNHYLSTQSVLSKDPNEK
$mNeonGreen3Asp_{1\text{-}10}$	MVSKGEEDNMASLPATHELHIFGSINGVDFDMVGQGTGNPNDGYEEL NLKSTKGDLQFSPWILVPHIGYGFHQYLPYPDGMSPFQAAMVDGSGY QVHRTMQFEDGASLTVNYRYTYEGSHIKGEAQVMGTGFPADGPVMT NTLTAADLCVSKMTYPNDKTIISTFKWSYTTVNGKRYRSTARTTYTFAK PMAAKYLKNQPMYVLRKTELKHSM
$sfCherry3Csp_{1\text{-}10}$	MEEDNMAIIKEFMRFKVHMEGSVNGHEFEIEGEGEGHPYEGTQTARL VVTKGDPLPFAWDILSPQFMYGSKAYVKHPADIPDYLKLSFPEGFTWE RVMNFEDGGVVYVTQDSSLQDGQFIYKVKLLGINFPSDGPVMQKKTM GWEASTERMYPEDGALKGEINQRLKLKDGGHYDAEVKTTYRAKKPVQ LPGPYDVDIKLDITSHNED
ΔSUMOstar	MTHINLKVSDGSSEIFFKIKKTTPLRRLMEAFAKRQGKEMDSLTFLYDGI EIQADQTPEDLDMEDNDIIEAHREQ
$CFP2_{11}$	RDHMVLHEYVNAAGIT
$mNeonGreen3Asp_{11}$	TELNFKEWQKAFTDD
$sfCherry3Csp_{11}$	TTIVEQYERAEARHTT
