# Supplementary material for: Intensity modulation of trichromatic split fluorescent proteins for live cell mapping
Source: Cell Rep Methods. 2026 Mar 26;6(4):101363. doi: 10.1016/j.crmeth.2026.101363 (PMC13107049; doi:10.1016/j.crmeth.2026.101363)
Supplement: Document S2. Article plus supplemental information [file mmc3.pdf]

Cell Reports
Methods

Article

Intensity modulation of trichromatic split fluorescent proteins for live cell mapping

Graphical abstract

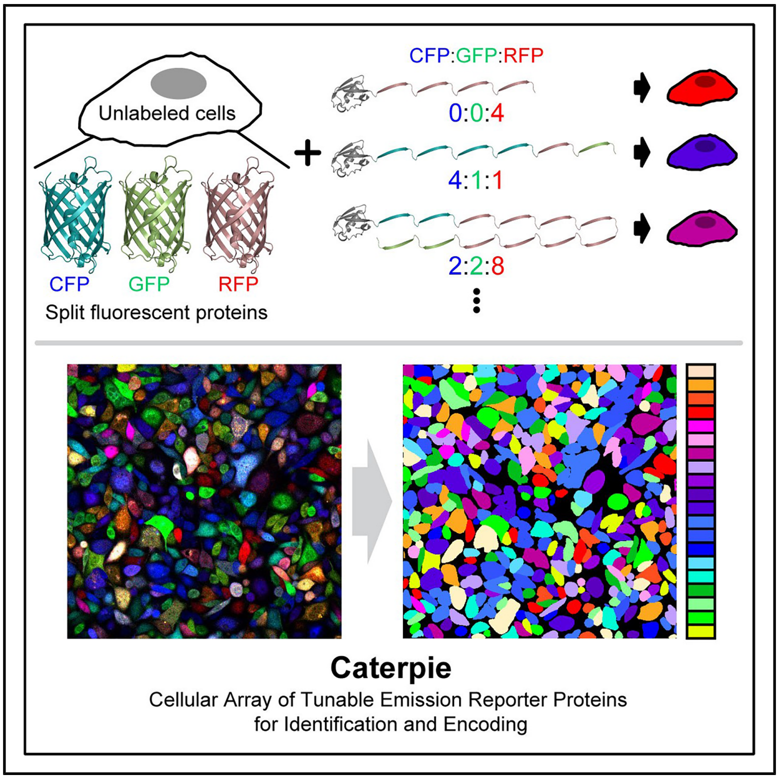

Authors

Mamoru Ishii, Tomoaki Kinjo, Yohei Kondo, Kenta Terai, Kazuhiro Aoki, Brian Kuhlman, Michiyuki Matsuda

Correspondence

mishii@iis.u-tokyo.ac.jp (M.I.),
matsuda.michiyuki.87r@st.kyoto-u.ac.jp (M.M.)

In brief

Ishii et al. present Caterpie, a rationally designed cell-labeling platform using engineered split fluorescent proteins. By modulating color and intensity combinations, Caterpie achieves precise identification of 20 distinct live-cell populations with 97% accuracy, enabling high-fidelity mapping and analysis of complex multicellular interactions.

Highlights

- Trichromatic split fluorescent proteins enable multiplexed live-cell labeling
- Structure-guided design improves brightness of mNG3A and sfCherry3C variants
- Intensity modulation allows reliable discrimination of 20 cell populations

Ishii et al., 2026, Cell Reports Methods *6*, 101363
April 20, 2026 © 2026 The Author(s). Published by Elsevier Inc.
https://doi.org/10.1016/j.crmeth.2026.101363

CellPress

Cell Reports Methods

CellPress
OPEN ACCESS

Article

Intensity modulation of trichromatic split fluorescent proteins for live cell mapping

Mamoru Ishii,[1,2,*] Tomoaki Kinjo,[3] Yohei Kondo,[4,5] Kenta Terai,[6] Kazuhiro Aoki,[1] Brian Kuhlman,[3,7] and Michiyuki Matsuda[1,8,9,10,*]
[1]Graduate School of Biostudies, Kyoto University, Sakyo-ku, Kyoto 606-8501, Japan
[2]Institute of Industrial Science, The University of Tokyo, Tokyo, Japan
[3]Department of Biochemistry and Biophysics, University of North Carolina School of Medicine, Chapel Hill, NC, USA
[4]Center for One Medicine Innovative Translational Research (COMIT), Nagoya University, Nagoya, Aichi, Japan
[5]Graduate School of Medicine, Nagoya University, Tsurumai-cho, Nagoya, Aichi 466-8550, Japan
[6]Graduate School of Medicine, Tokushima University, Shinkura-cho, Tokushima 770-8501, Japan
[7]Lineberger Comprehensive Cancer Center, University of North Carolina at Chapel Hill, Chapel Hill, NC, USA
[8]Affiliate Graduate School, Graduate School of Medicine, Kyoto University, Sakyo-ku, Kyoto 606-8501, Japan
[9]Integrated Graduate School of Medicine, Engineering, and Agricultural Sciences, University of Yamanashi, Chuo-shi, Yamanashi 409-3898, Japan
[10]Lead contact
*Correspondence: mishii@iis.u-tokyo.ac.jp (M.I.), matsuda.michiyuki.87r@st.kyoto-u.ac.jp (M.M.)
https://doi.org/10.1016/j.crmeth.2026.101363

MOTIVATION Existing fluorescent protein-based multiplexed labeling systems rely on stochastic color selection, which limits the ability to accurately identify specific cell populations. Moreover, expanding spectral diversity by concatenating multiple fluorescent proteins increases gene size and complicates expression. These challenges hinder reliable discrimination of multiple defined cell populations in live imaging.

SUMMARY

Current fluorescent protein-based multiplexed cell labeling techniques suffer from limited discrimination power due to stochastic color selection and large gene sizes from tandem repeats of multiple fluorescent proteins. We developed Caterpie, a rationally designed system using engineered split fluorescent proteins that enables deterministic identification of 20 distinct cell populations with 97% accuracy and reduced gene sizes. Through computational structure-guided design, we engineered enhanced split mNeonGreen3A and split sfCherry3C variants that achieve performance comparable to split CFP2, the best-performing split fluorescent protein. Our systematic library of trichromatic 11th β-strand tags with up to 12 tandem repeats enables predictable, high-fidelity labeling for precise cell targeting. This technology addresses critical limitations in simultaneous identification of multiple defined cell populations.

INTRODUCTION

Multiplexed spectral labeling techniques represent essential tools across diverse biological disciplines, including neuroscience, developmental biology, and immunology. These techniques enable critical applications such as discrimination of adjacent cells, labeling of specific cell populations, and lineage tracing. The Brainbow system exemplifies this approach, utilizing multiple gene cassettes designed to stochastically express one of three fluorescent proteins (FPs), thereby generating distinct spectral signatures.[1] By increasing the number of fluorescent protein genes to five, the development of Bitbow system helped achieve up to 31 ($2^5 - 1$) unique color combinations.[2] In addition to increasing the number of fluorescent protein genes, another promising avenue for expanding labeling capacity involves harnessing fluorescence intensity information. By utilizing intensity ratios between different FPs (such as GFP:RFP ratios of 1:1, 1:2, or 2:1), the number of distinguishable cellular labels can be substantially increased. However, approaches that expand color diversity through serial concatenation of multiple FPs face practical constraints due to increasing gene sizes. Split fluorescent proteins (split FPs) emerge as an elegant solution to these technical challenges.

Split FPs are generated through strategic dissection of β-barrel FPs into two components: the 11th β-strand (FP_{11}) and a complementary segment comprising the first ten β-strands (FP_{1-10}).[3] While neither fragment exhibits fluorescence independently, their co-expression facilitates spontaneous reassembly, enabling chromophore maturation and subsequent fluorescence emission. This complementation system has been successfully

© 2026 The Author(s). Published by Elsevier Inc.
This is an open access article under the CC BY license (http://creativecommons.org/licenses/by/4.0/).

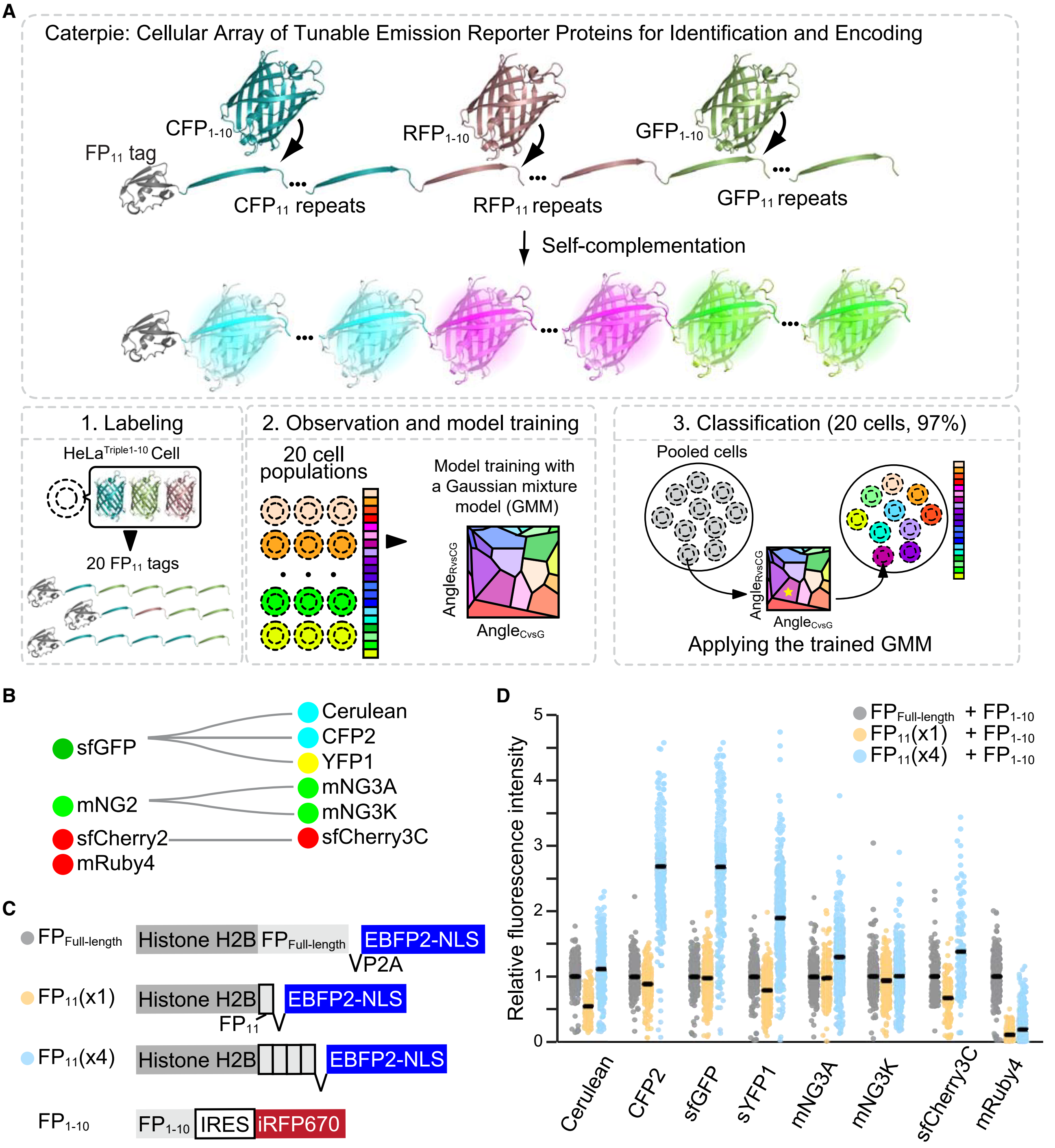

Figure 1. Caterpie: A cell identification system based on split fluorescent protein arrays

(A) Schematic of the Caterpie approach, illustrating cell identification through modular arrays of split fluorescent proteins.

(B) Evolutionary relationships among split fluorescent proteins utilized in this study, presented as a phylogenetic tree.

(C) Molecular architecture of expression constructs: Histone H2B fusions containing either full-length FP ($FP_{Full\text{-}length}$), single FP_{11} fragment, or tetrameric FP_{11} arrays, shown alongside the complementary $FP_{1–10}$ fragment.

(D) Quantitative analysis of relative fluorescence intensity in HeLa cells expressing $FP_{Full\text{-}length}$, $FP_{11}(\times1)$, or $FP_{11}(\times4)$, with $FP_{1–10}$ fragments. $FP_{1–10}$ was transfected at 4-fold excess to ensure sufficient intracellular availability for optimal complementation with FP_{11}. Cells were observed under a confocal

(legend continued on next page)

employed for protein labeling applications, wherein target proteins are tagged with FP_{11} in cells expressing $FP_{1–10}$.[4,5] A particularly advantageous feature of this system emerges under conditions of abundant $FP_{1–10}$ expression: enhanced fluorescence intensity can be achieved by increasing the number of FP_{11} tags conjugated to the protein of interest, while maintaining relatively modest gene size requirements.[6–9]

Drawing inspiration from human color perception, which discriminates diverse spectral signatures through the integration of three primary color intensities—blue, green, and red—we developed a multiplexed cellular labeling approach. This system exploits engineered arrays of FP_{11} fragments derived from cyan, green, and red split FPs, each designed to complement with their cognate $FP_{1–10}$ fragments. Our method achieves robust discrimination of 20 distinct cell populations, with 97% accuracy using epi-fluorescence microscopy.

RESULTS

Split FPs for the trichromatic color palette

We aimed to establish a trichromatic color palette through the systematic engineering of tandem FP_{11} repeats derived from cyan, green, and red split FPs, which undergo fluorescence complementation when co-expressed with their cognate $FP_{1–10}$ fragments (Figure 1A). For this, we sought to select split FPs that achieve brightness comparable to their full-length FP ($FP_{Full\text{-}length}$) and exhibit fluorescence signal amplification through FP_{11} repeats. A critical consideration in designing this system is the requirement for sequence divergence among the trichromatic split FPs to prevent cross-complementation between fragments from different fluorescent protein pairs.[10] To address this constraint, we evaluated split FPs derived from distinct evolutionary lineages: sfGFP, mNeonGreen2 (mNG2), sfCherry2, and mRuby4, though the latter two systems show some cross-complementation (Figure 1B). To quantitatively assess the relative brightness of $FP_{Full\text{-}length}$, $FP_{11}(\times1)$, and $FP_{11}(\times4)$ of the reported split FPs, we developed a dual-reporter system. We engineered expression constructs encoding histone H2B-tagged $FP_{Full\text{-}length}$, $FP_{11}(\times1)$, or $FP_{11}(\times4)$ fused to EBFP2-nls (nuclear localization signal) through a self-cleaving P2A site, enabling normalization of expression levels through nuclear EBFP2 fluorescence. These constructs were co-transfected into HeLa cells with a secondary plasmid encoding $FP_{1–10}$ linked to iRFP670 via an internal ribosome entry site (IRES) at a DNA ratio of 1:4 (Figure 1C). To prevent potential CMV promoter competition and dilution effects, total DNA amounts and the ratio of expression plasmids were kept consistent across all transfection conditions.

After 48 h, cells were observed under a confocal microscope, and the fluorescence intensities of split FPs were quantified in iRFP670-positive cells (Figure 1D). For each FP variant, relative fluorescence intensity values were normalized to the $FP_{Full\text{-}length}$. In some cases, $(\times1)$ showed decreased fluorescence brightness compared to $FP_{Full\text{-}length}$, but sfGFP, mNG3A, and mNG3K showed almost equivalent fluorescence brightness. Comparative analysis of $(\times1)$ versus $(\times4)$ constructs revealed that all sfGFP-derived split FPs exhibited more than 2-fold enhancement in median brightness, with CFP2 demonstrating the most substantial increase of more than 3-fold. Consistent with previous findings,[11] split mNG3A demonstrated superior amplification from $(\times1)$ to $(\times4)$ compared with split mNG3K. Split $sfCherry3C_{11}(\times1)$ exhibited diminished fluorescence intensity relative to $sfCherry3C_{Full\text{-}length}$, attributable to reduced association efficiency, corroborating earlier observations.[12] The truncated $mRuby4_{11}$ showed markedly decreased fluorescence intensity, highlighting the critical role of its C-terminal unstructured polypeptide chain.[10] These preliminary findings indicated that while the combination of CFP2, mNG3A, and sfCherry3C provides a promising foundation for the trichromatic system, further engineering optimization of mNG3A and sfCherry3C is necessary to achieve optimal performance across all spectral channels.

Fluorescence intensity-based cell classification using tandem $CFP2_{11}$ β-strand repeats

Before the engineering of mNG3A and sfCherry3C, we established a platform for cell identification through fluorescence intensity modulation by using $CFP2_{11}$ repeats. To this end, we constructed plasmids encoding repeated sequences of $CFP2_{11}$ (Figure 2A). Initially, we engineered a plasmid encoding a histone H2B-$CFP2_{11}(\times1)$ fusion construct, in which the $CFP2_{11}(\times1)$ sequence was flanked by a BglII restriction site at its 5′ terminus and BamHI and NotI restriction sites at its 3' terminus. Through sequential ligation of BglII/NotI-digested fragments (insert) with BamHI/NotI-digested fragments (vector), we successfully amplified $CFP2_{11}$ to create $(\times2)$, $(\times4)$, and $(\times8)$ variants. Based on previous research,[6] we incorporated GGSGG linker sequences between $CFP2_{11}$ fragments. At the BglII-BamHI junction, the six nucleotides encode glycine and serine residues, thereby serving as an integral part of the linker sequence. The $CFP2_{11}$ repeats ([×1], [×2], [×4], or [×8]) fused with histone H2B were introduced into HeLa cells stably expressing $CFP2_{1\text{-}10}$ ($HeLa^{CFP2\ 1\text{-}10}$) (Figure 2B). As a control, we expressed histone H2B-tagged $CFP2_{Full\text{-}length}$ in the same $HeLa^{CFP2\ 1–10}$ cell line. When normalized to nuclear mCherry fluorescence, the CFP2 fluorescence intensity increased across consecutive constructs with a factor of approximately 1.7-fold, which was slightly lower than the theoretically expected 2-fold increment (Figure 2C).

To identify cells expressing different numbers of $CFP2_{11}$ repeats, we implemented a Gaussian mixture model (GMM) for cell classification (Figure 2D). The fluorescence intensities of mCherry and CFP2 were transformed into polar coordinates, where the $Angle_{mCherry\ vs\ CFP2}$ (ranging from 0° to 90°)

microscope and analyzed as follows: First, the fluorescence intensity ratio of $FP_{Full\text{-}length}$ to EBFP was calculated for each cell expressing $FP_{Full\text{-}length}$, and the median of these ratios was determined as a normalization standard. Next, for cells positive for iRFP670 (confirming successful FP1–10 expression), the ratio of nuclear split-FP fluorescence to EBFP fluorescence was calculated. Finally, these split-FP/EBFP ratios were divided by the median value obtained in the first step to yield relative fluorescence intensities. Data presented as bee swarm plots, with median values indicated by black lines (n > 130 cells per condition).

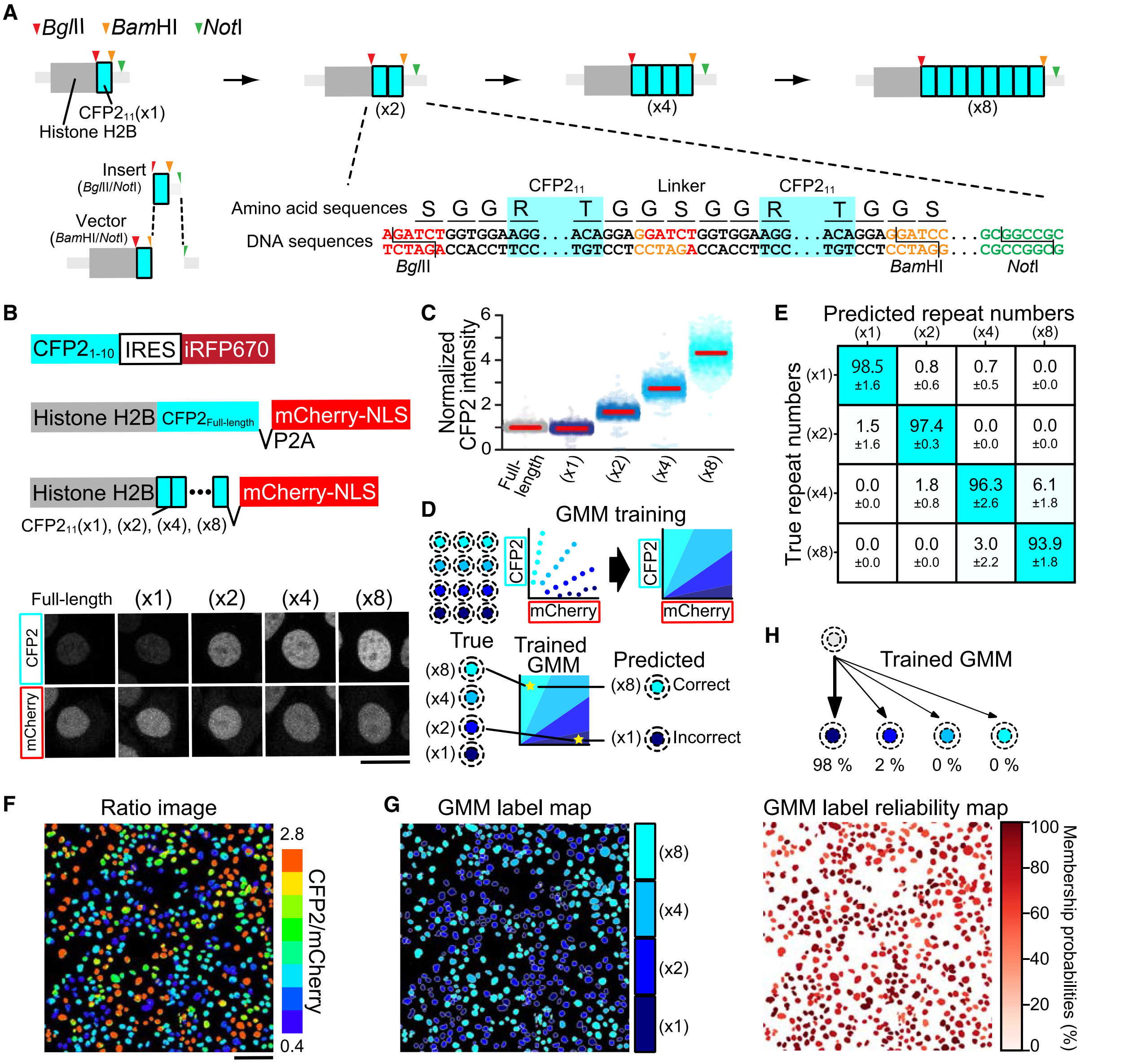

Figure 2. Signal amplification and cell classification using split CFP2 arrays

(A) Molecular strategy for generating tandem CFP2$_{11}$ repeats, including detailed amino acid and nucleotide sequences at the CFP2$_{11}$(×1) insert-vector junction.

(B) Construct architecture and expression analysis. Top: Schematics for CFP2$_{1-10}$ and histone H2B fusions containing either full-length CFP2 (CFP2$_{Full-length}$) or varying copy numbers of CFP2$_{11}$ [CFP2$_{11}$(×1), (×2), (×4), and (×8)]. Bottom: Representative confocal micrographs of HeLa cells stably co-expressing CFP2$_{1-10}$ with either CFP2$_{Full-length}$ or CFP2$_{11}$ variants. Scale bars, 20 μm.

(C) Quantitative analysis of normalized CFP2 fluorescence in HeLa cells expressing histone H2B-tagged CFP2$_{11}$ [(×1), (×2), (×4), or (×8)]. Data are presented as bee swarm plots with median values (red lines); >1,300 cells were analyzed across three independent experiments.

(D) Workflow schematic for Gaussian mixture model (GMM) and implementation.

(E) Classification performance matrix showing prediction accuracy against true labels. Data are represented as the mean ± SD of prediction accuracy from three independent experiments, with color intensity indicating classification accuracy.

(F) Representative ratio image of pooled cells expressing different CFP2$_{11}$ copy number variants [(×1), (×2), (×4), or (×8)]; scale bars: 100 μm.

(G) Cell classification map derived from (F), showing GMM-based assignment of individual cells to specific copy number variants [(×1), (×2), (×4), or (×8)].

(H) Visualization of GMM classification confidence through membership probability mapping of cells shown in (G).

represented the CFP2/mCherry intensity ratio. The angular data were divided into training and test datasets. We trained a GMM on the training dataset to establish classification parameters, determining the decision boundary of the Angle$_{mCherry\ vs\ CFP2}$. The model performance was validated using the test dataset, and the predicted repeat numbers were compared with the

CellPress
OPEN ACCESS

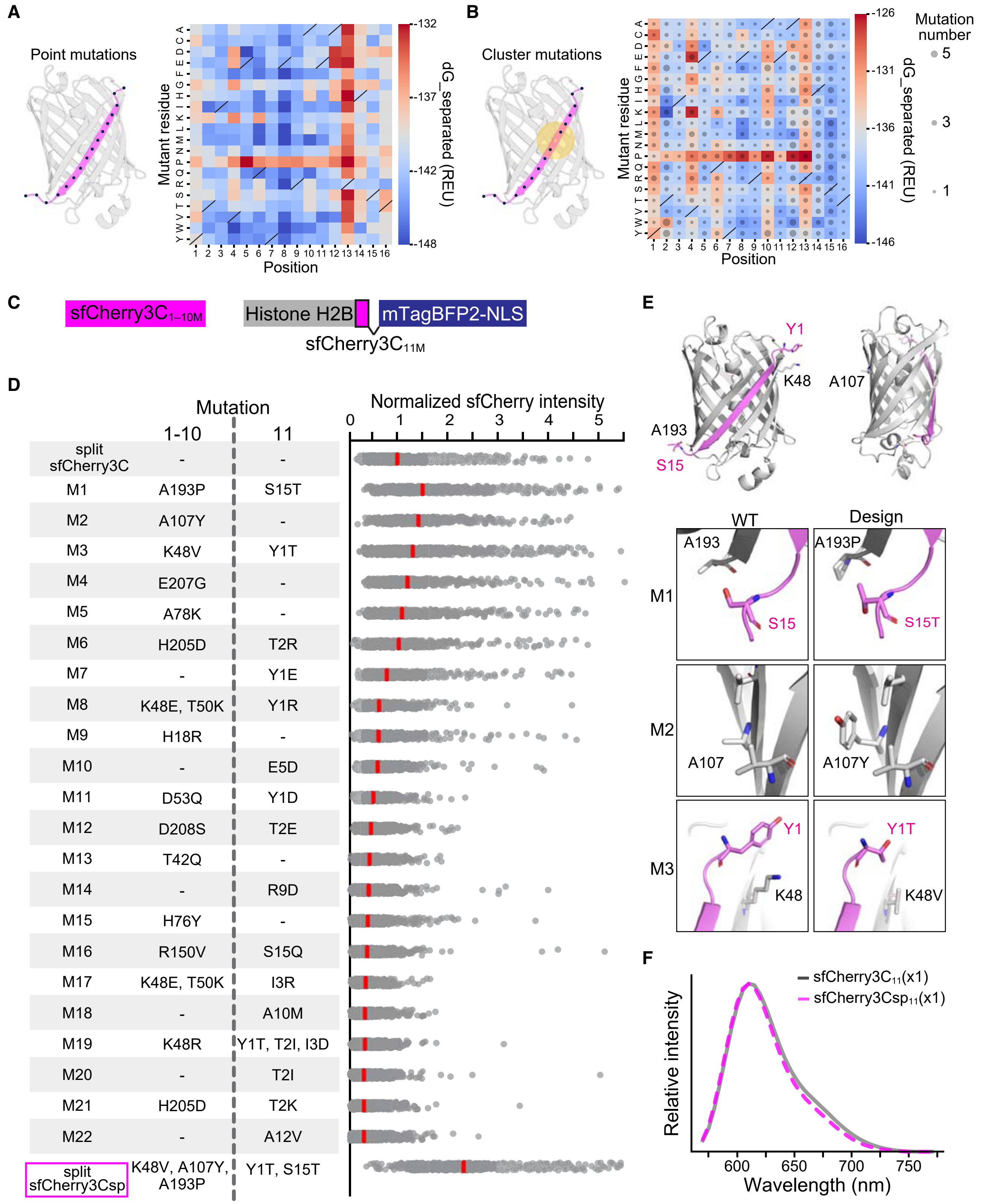

(legend on next page)

actual repeat numbers. The model achieved an overall accuracy of 96% (Figure 2E). Among all constructs, CFP2$_{11}$(×8) exhibited the lowest accuracy (93.9%), with 6.1% of (×8)-expressing cells being misclassified as (×4).

To validate the system's discriminative capacity in a heterogeneous context, we analyzed mixed populations of cells expressing four different CFP2$_{11}$ repeat variants by using fluorescence microscopy and generated CFP2/mCherry ratio images (Figure 2F). The trained GMM demonstrated robust performance in classifying individual cells into (×1), (×2), (×4), or (×8) populations (Figure 2G). The GMM provided membership probabilities for each cell—quantitative confidence scores indicating how likely each cell belongs to a specific population—enabling quantitative assessment of classification reliability (Figure 2H). These results established that four-level intensity modulation of a single FP is sufficient for reliable population discrimination.

Computational design of split sfCherry3C for enhanced β-barrel stability and split-strand complementation

To optimize split sfCherry3C toward fluorescence intensities close to those of the full-length protein, we modeled the sfCherry3C$_{1\text{-}10}$/sfCherry3C$_{11}$ complex with ColabFold[13] (based on AlphaFold-Multimer)[14] and used the structure model for subsequent design with the Rosetta modeling suite.[15] Because both global β-barrel stability and $\beta_{1\text{-}10}$/β_{11} interface affinity are crucial for fluorescence of split FPs, we pursued two design strategies in parallel. First, to enhance global β-barrel stability, we performed *in silico* site-saturation mutagenesis (SSM)[16] across all 224 residues, ranking each variant by the change in calculated energy (ΔE), as measured with the Rosetta force field (Figure S1). Next, to refine the β_{1-10}-β_{11} interface, we repurposed the SSM script for interface design; with the revised script, we performed SSM at every β_{11} position and calculated the binding energy of each single mutant with Rosetta's InterfaceAnalyzer protocol (Figure 3A). To create FP variants with multiple mutations, each single-mutant model served as the starting point for a design simulation in which residues within 5–7 Å of the point mutation were allowed to mutate and adopt alternative conformations.[16] Designs calculated to have favorable protein energies and binding energies were visually inspected to identify variants that reinforce interface contacts, while excluding ones that disrupted the chromophore-forming triad M67–Y68–G69 or introduced additional aromatic residues into β_{11}, which could promote aggregation of the multimeric β_{11} repeat. Following these criteria, we selected 22 candidate designs for subsequent validation in cells.

Upon stable co-expression of β_{1-10} and β_{11} in HeLa cells, three variants (M1, M2, and M3) outperformed the parental split sfCherry3C in fluorescence intensity (Figures 3C and 3D). We then combined the mutations from the three top-performing variants to generate an optimized variant, named split sfCherry3Csp, which achieved a 2.5-fold enhancement in fluorescence intensity compared to the parental split sfCherry3C. M1 and M3 mutations locate at the β_{1-10}-β_{11} interface, where the models suggest the improved association of β_{1-10} and β_{11} through the formation of reinforced side-chain contacts. The M2 mutation lies on the opposite face of the interface, implying an effect on β-barrel stability (Figure 3E). The fluorescence spectra of split sfCherry3Csp were indistinguishable from those of the parental split sfCherry3C (Figure 3F).

Enhancement of complementation efficiency through structure-guided engineering of split mNG3A

As shown in Figure 1D, split mNG3A demonstrated only modest signal amplification, with a 1.3-fold increase in fluorescence intensity from (×1) to (×4) variants. To gain insight into the structural factors underlying this limitation, we modeled the octameric mNG3A$_{11}$ peptide by using ColabFold[13] (based on AlphaFold2).[17] The predicted structures suggested that the mNG3A$_{11}$ repeat adopts a loosely helical conformation, with hydrophobic residues (F5, W8, F12, M15, and M16) converging to form continuous hydrophobic interactions, despite only moderate model confidence. These features implied that multimeric mNG3A$_{11}$ may self-aggregate via its hydrophobic surfaces (Figure 4A). Additionally, inspection of the parental mNG crystal structure (PDB: 5LTP) and the structure model of split mNG3A suggested that the C-terminal residues D14–M15–M16 contribute minimally to the β-barrel fold (Figure 4B). We, therefore, reasoned that removing these C-terminal residues could mitigate the aggregation propensity without perturbing β-barrel and chromophore maturation. Guided by these insights, we implemented three C-terminal modification approaches: (1) deletion of the C-terminal residues D14-M15-M16, (2) addition of one or two aspartic acid residue to preserve charge that could electrostatically repel neighboring fragments, and (3) substitution of F12 with tyrosine to introduce polarity (Figure 4C). To evaluate these modifications, we expressed 8 repeats of the

Figure 3. Computational design of split sfCherry3C

(A) *In silico* site-saturation mutagenesis of sfCherry3C$_{11}$. Left: Schematic of the β_{11} residues targeted for saturation mutagenesis (black spheres). Right: Heatmap of mean dG_separated values in Rosetta energy unit (REU) across three independent models for each amino acid substitution at each position. Black slashes indicate wild-type amino acids.

(B) Cluster-mutation design of sfCherry3C$_{11}$. Left: Schematic showing a representative seed point mutation (yellow dot) and the shell of neighboring residues (yellow spheres) targeted for sequential cluster mutagenesis. Right: Heatmap of the mean dG_separated value across ten cluster-mutated models derived from each seed mutation at every β_{11} site; dot size indicates the median number of cluster-mutated residues across these ten models. Black slashes indicate wild-type amino acids.

(C) Schematics of mutant variants of sfCherry3C$_{1\text{-}10}$ (sfCherry3C$_{1-10M}$) and histone H2B-tagged mutant variant of sfCherry3C$_{11}$ (sfCherry3C$_{11M}$).

(D) List of mutant variants of sfCherry3C$_{1-10M}$ and sfCherry3C$_{11M}$. Bee swarm plot showing normalized sfCherry3C fluorescence intensity of HeLa cells stably co-expressing histone H2B-tagged sfCherry3C$_{11M}$(×1) with sfCherry3C$_{1-10M}$. Red lines represent the median.

(E) Rosetta models of split sfCherry3Csp. The overall β-barrel is shown as a cartoon colored by fragment (gray, $\beta_{1\text{-}10}$; magenta, β_{11}). Each inset zooms on an engineered site, showing the parental conformation on the left and the corresponding design on the right, with key residues rendered as stick representation. All models were visualized in PyMOL.

(F) Emission spectra of HeLa cells expressing split sfCherry3C or split sfCherry3Csp with 546 nm laser excitation.

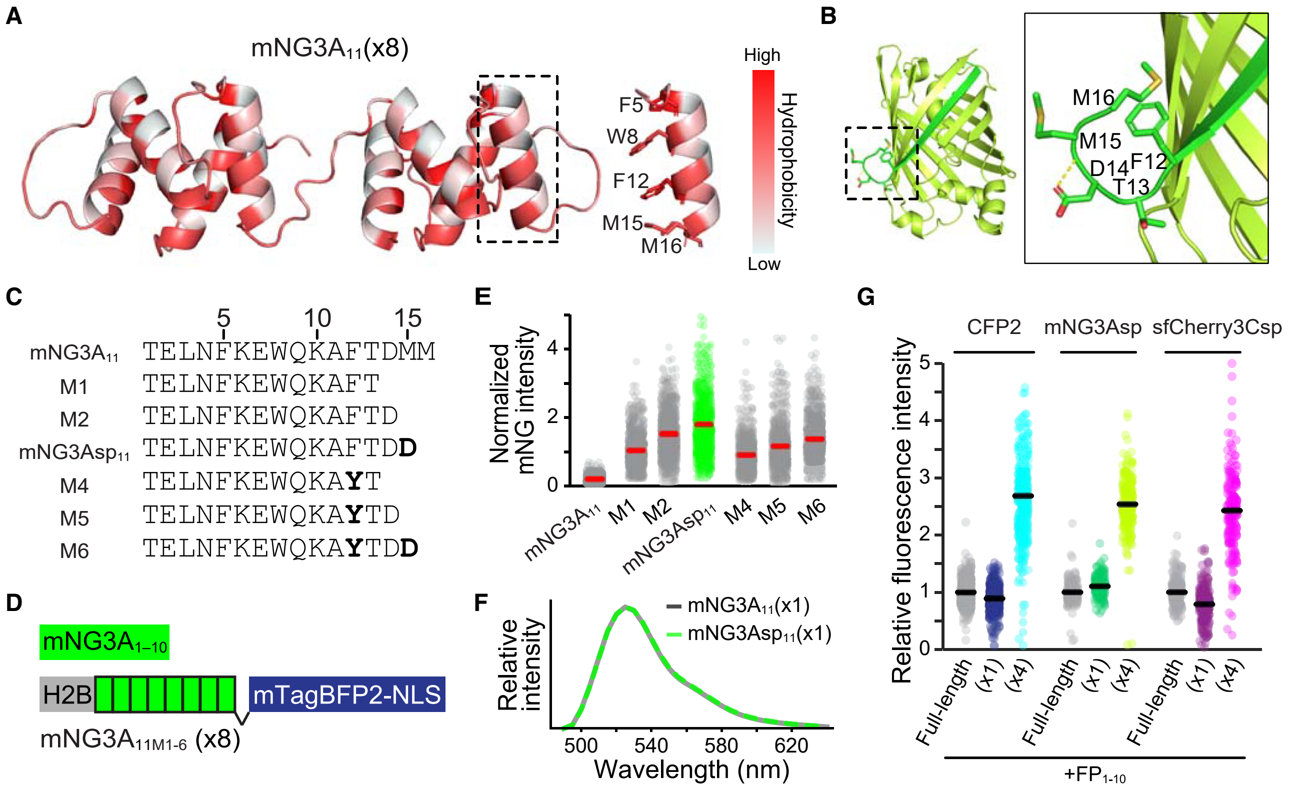

Figure 4. Optimization of split mNG3A through structure-guided C-terminal engineering

(A) Predicted structural model of native mNG3A$_{11}$($\times$8) generated using ColabFold (AlphaFold2) and visualized with PyMOL. Colors represent hydrophobicity according to the Eisenberg hydrophobicity scale.

(B) Predicted structural model of parental split mNG3A generated using ColabFold (AlphaFold Multimer) and visualized with PyMOL. Color-coded domains: mNG3A$_{1\text{-}10}$ (light green) and mNG3A$_{11}$ (green).

(C) Comparative sequence analysis of original mNG3A$_{11}$ and engineered mutant variants (mNG3A$_{11M}$).

(D) Schematic of the expression constructs: mNG3A$_{1\text{–}10}$ and histone H2B fusion with the mNG3A$_{11M}$($\times$8) array.

(E) Quantitative analysis of normalized mNG3A fluorescence intensity in HeLa cells stably co-expressing histone H2B-tagged mNG3A$_{11M}$ ($\times$8) with mNG3A$_{1\text{–}10}$. Median values are indicated by red lines. Values were normalized to set the median value of mNG3A$_{11M1}$($\times$8) to 1.

(F) Emission spectra of HeLa cells expressing split mNG3A or split mNG3Asp with 470 nm laser excitation.

(G) Comparative analysis of the relative fluorescence intensity of HeLa cells expressing FP$_{\text{Full-length}}$, FP$_{11}$($\times$1) or FP$_{11}$($\times$4), with cognate FP$_{1\text{–}10}$ fragments (1:4 transfection ratio). Data are presented as bee swarm plots with median values (black lines); $n > 130$ cells per condition. Measurements were obtained through confocal microscopy.

mNG3A$_{11}$ fragments fused to histone H2B in HeLa cells expressing mNG3A$_{1\text{–}10}$ (Figure 4D). Nuclear mNG3A fluorescence was quantified through microscopy, with expression levels normalized to nuclear mTagBFP2 fluorescence intensity (Figure 4E). Among the modifications tested, we chose the mNG3Asp$_{11}$ variant for further analysis because of its highest fluorescence intensity, which was 9.1-fold brighter than that of the parental mNG3A$_{11}$. The fluorescence spectrum obtained using the mNG3Asp$_{11}$ fragment was identical to that observed with the mNG3A$_{11}$ fragment (Figure 4F).

Performance evaluation of split mNG3Asp and split sfCherry3Csp was conducted in parallel with split CFP2, following our previously established experimental framework (Figure 4G). Quantitative analysis revealed that mNG3Asp$_{11}$($\times$4) achieved a 2.3-fold enhancement in fluorescence intensity compared to its monomeric counterpart. The engineered sfCherry3Csp demonstrated robust performance, with its monomeric variant exhibiting 80% of the fluorescence intensity of the full-length protein, while the tetrameric construct showed a 3-fold signal amplification relative to the monomer. These significant improvements in signal amplification efficiency indicate that both engineered variants, split mNG3Asp and split sfCherry3Csp, now achieve performance metrics comparable to split CFP2, providing the basis for trichromatic imaging.

Selection of 20 optimal FP11 tags with optimized fusion partner

We expect to use lentivirus as the vector, where packaging capacity limits the size of the cDNA insert. Therefore, we aimed to replace the nuclear-localized histone H2B with smaller protein tags. We compared histone H2B (126 amino acids) with two smaller alternatives that are known to improve the stability and solubility of fusion proteins: GB1 (56 amino acids)[18] and ΔSUMOstar (74 amino acids), a truncated version of

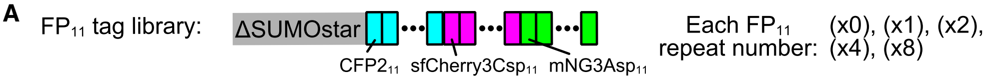

ID	1	2	3	4	5	6	7	8	9	10	11	12	13	14	15	16	17	18	19	20	21	22	23	24	25	26	27	28	29	30	31	32
C	1	2	4	8	0	0	0	0	0	0	0	0	1	1	1	1	2	2	2	2	4	4	4	4	8	8	8	1	1	1	1	2
G	0	0	0	0	1	2	4	8	0	0	0	0	1	2	4	8	1	2	4	8	1	2	4	8	1	2	4	0	0	0	0	0
R	0	0	0	0	0	0	0	0	1	2	4	8	0	0	0	0	0	0	0	0	0	0	0	0	0	0	0	1	2	4	8	1
1st			O			O		O		O		O				O		O	O						O						O	
2nd			O			O						O				O		O	O						O							
Final				O				O			O					O		O	O						O							

ID	33	34	35	36	37	38	39	40	41	42	43	44	45	46	47	48	49	50	51	52	53	54	55	56	57	58	59	60	61	62	63	64
C	2	2	2	4	4	4	4	8	8	8	0	0	0	0	0	0	0	0	0	0	0	0	0	0	0	1	1	1	1	1	1	1
G	0	0	0	0	0	0	0	0	0	0	1	1	1	1	2	2	2	2	4	4	4	4	8	8	8	1	1	1	1	2	2	2
R	2	4	8	1	2	4	8	1	2	4	1	2	4	8	1	2	4	8	1	2	4	8	1	2	4	1	2	4	8	1	2	4
1st		O		O														O				O	O	O	O		O					O
2nd		O		O														O				O		O								O
Final		O		O														O				O		O								O

ID	65	66	67	68	69	70	71	72	73	74	75	76	77	78	79	80	81	82	83	84	85	86	87	88	89	90	91	92	93	94	95	96
C	1	1	1	1	1	1	2	2	2	2	2	2	2	2	2	2	2	2	2	4	4	4	4	4	4	4	4	4	8	8	8	8
G	2	4	4	4	8	8	1	1	1	1	2	2	2	2	4	4	4	8	8	1	1	1	2	2	2	4	4	4	1	1	2	2
R	8	1	2	4	1	2	1	2	4	8	1	2	4	8	1	2	4	1	2	1	2	4	1	2	4	1	2	4	1	2	1	2
1st				O	O	O	O	O						O						O		O		O				O			O	
2nd				O		O		O						O						O								O			O	
Final				O		O		O						O						O								O			O	

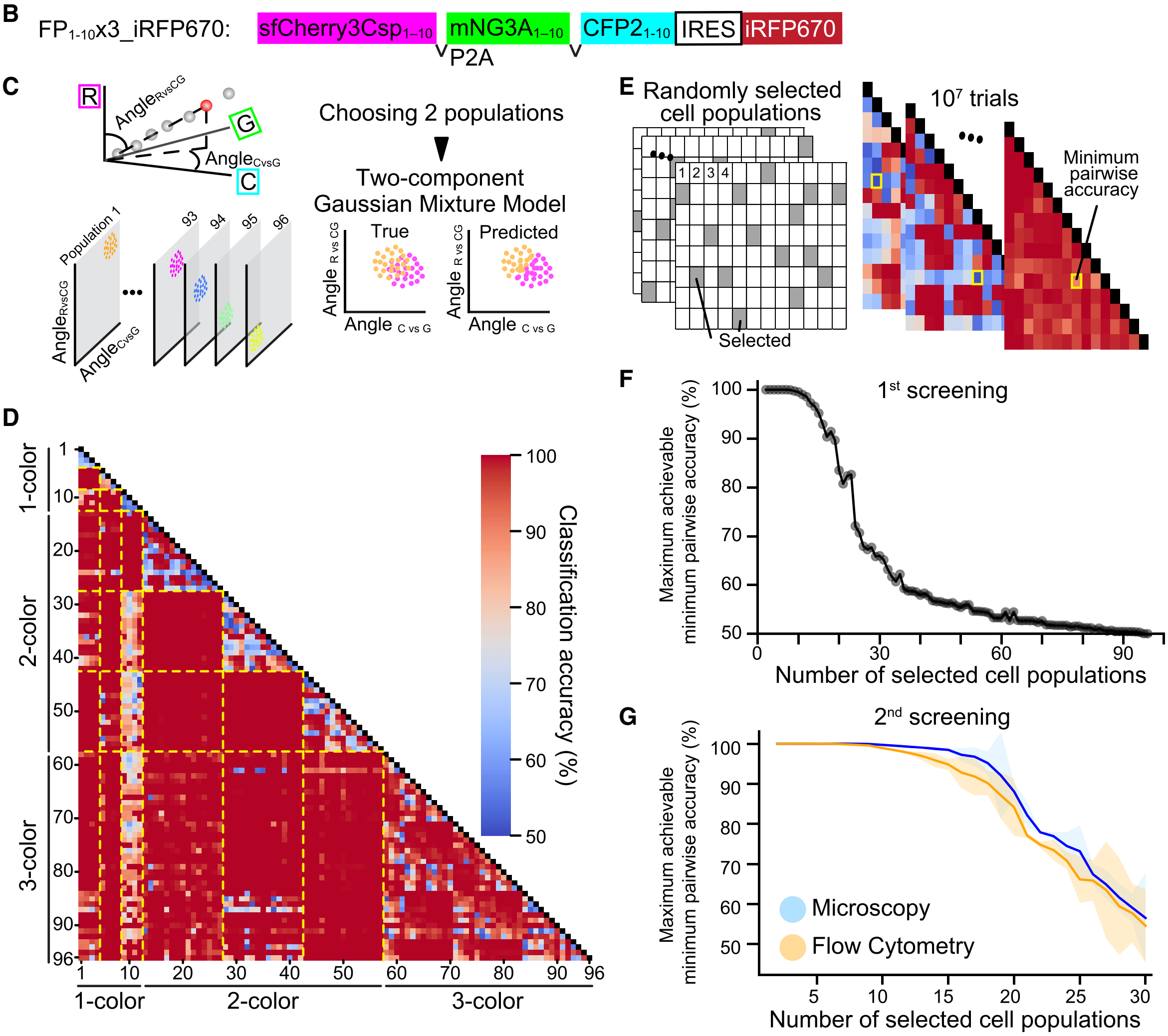

(legend on next page)

SUMOstar,[19] as well as constructs lacking fusion tags (Figure S2A). ΔSUMOstar was engineered by removing the unstructured polypeptide regions from both termini of SUMOstar; the resulting sequence is detailed in Figure S2B. In agreement with previous studies,[20] constructs lacking fusion proteins demonstrated markedly reduced fluorescence intensity—an effect that persisted even in the octameric (×8) variant (Figure S2C). Comparative analysis of fusion partners revealed that ΔSUMOstar-tagged constructs achieved substantially higher fluorescence intensity than GB1-tagged variants in octameric configurations, demonstrating the importance of fusion partner selection for optimal signal amplification. The ΔSUMOstar-tagged constructs exhibited consistent signal enhancement, with fluorescence intensity increasing 1.6-fold for each doubling of $CFP2_{11}$ repeats, ultimately enabling 93% discrimination accuracy between variants (Figures S2D–S2F).

We next examined whether the sequential order of the three FP_{11} variants ($CFP2_{11}$, $mNG3Asp_{11}$, and $sfCherry3Csp_{11}$) may change the efficiency of the complementation with FP_{1-10} fragments. For this, we generated ΔSUMOstar fusion constructs containing six total repeats, wherein each FP_{11} fragment was represented in duplicate (Figure S2G). The complete set of six possible permutations was generated and stably expressed in HeLa cells constitutively expressing all three FP_{1-10} fragments. Quantitative analysis of the relative fluorescence intensities was performed using EBFP2 fluorescence for normalization of expression levels (Figure S2H). Among all configurations evaluated, the $CFP2_{11}$(×2)-$sfCherry3Csp_{11}$(×2)-$mNG3Asp_{11}$(×2) arrangement demonstrated marginally superior fluorescence intensity across all spectral channels, leading to its selection for subsequent studies.

Based on these preliminary findings, we established a comprehensive library of fusion constructs combining ΔSUMOstar with $CFP2_{11}$, $sfCherry3Csp_{11}$, and $mNG3Asp_{11}$ fragments in this order (Figure 5A). Each fragment was represented in varying copy numbers ([×0], [×1], [×2], [×4], or [×8]) for each. In the first screening, we limited the FP_{11} tag combinations to 96 variants, with the total number of repeats not exceeding (×12). The integrity of all FP_{11} repeat sequences in the 96 plasmid constructs was verified by Sanger sequencing, thereby establishing a comprehensive FP_{11} tag library.

To generate a stable cellular platform for evaluating the FP_{11} tag library, we engineered an expression vector encoding all three FP_{1-10} fragments (CFP_{1-10}, $sfCherry3Csp_{1-10}$, and $mNG3A_{1-10}$) separated by self-cleaving P2A peptides, followed by an IRES and iRFP670 (Figure 5B). Through fluorescence-activated cell sorting (FACS) based on iRFP670 expression levels, we established a HeLa cell clone ($HeLa^{FP1-10x3\_iRFP670}$) expressing high levels of three FP_{1-10} fragments.

The complete library of 96 FP_{11} tags was stably expressed in $HeLa^{FP1-10x3\_iRFP670}$ cells, with resultant populations analyzed by confocal microscopy. To reduce the three-dimensional information of the fluorescence distribution patterns to the two-dimensional form, we implemented spherical coordinate transformation to calculate two angular parameters: $Angle_{CvsG}$ and $Angle_{RvsCG}$, each ranging from 0° to 90° (Figure 5C, left). To evaluate the discriminative power of these angular parameters, we performed pairwise population analysis by using selected cell populations from the library. Cell classification was achieved using an unsupervised, two-component GMM without prior training data (Figure 5C, right). Classification accuracy was determined by comparing model-predicted clusters against known population identities. Superior classification accuracy correlated with greater separation of populations in the $Angle_{CvsG}$ – $Angle_{RvsCG}$ parameter space. This analytical framework was systematically applied to all possible pairwise combinations within the 96 FP_{11}-tagged cell library (Figure 5D).

To determine the number of distinguishable cell populations, we implemented an iterative sampling approach due to the vast number of possible patterns (approximately 2×10^{20}) when selecting 20 FP_{11}-tagged cells from a pool of 96 (Figure 5E). Random subsets of predetermined sizes were drawn from the 96 FP_{11}-tagged cell populations, with pairwise classification accuracies extracted from the comprehensive analysis presented in Figure 5D. For each FP_{11}-tagged subset, we identified the minimum classification accuracy, reflecting the subset's ability to distinguish the most similar cell population pairs. This sampling process was repeated 10^7 times, and the maximum achievable minimum accuracy was plotted as a function of subpopulation size (Figure 5F). The analysis revealed that a subset of 20 cell populations maintained robust discrimination, with a minimum pairwise classification accuracy of 90%. However, expanding to 30 populations resulted in a substantial decrease in the minimum accuracy to 60%. Based on these quantitative insights, we selected an initial panel of 30 candidate FP_{11} tags (Figure 5A, 1st) for subsequent refinement to establish the 2nd optimized set of 20 tags (Figure 5A, 2nd) that would ensure maximal discriminative power.

Figure 5. Systematic selection and validation of FP_{11} tags for Caterpie implementation

(A) Schematics of FP_{11} tags: ΔSUMO-tagged FP_{11} arrays comprising paired repeats of $CFP2_{11}$, $mNG3Asp_{11}$, and $sfCherry3Csp_{11}$ [(×0), (×1), (×2), (×4), (×8) each]. Comprehensive catalog of the FP_{11} tag library comprising 96 distinct combinations, detailing copy numbers of $CFP2_{11}$ (labeled as "C"), $mNG3Asp_{11}$ (labeled as "G"), and $sfCherry3Csp_{11}$ (labeled as "R") fragments. The marks indicate the FP_{11} tags selected during primary and secondary screening processes and those finally chosen.

(B) Schematic of the expression constructs: $FP_{1-10} \times 3$.

(C) Analysis scheme for the classification of cell populations expressing various FP_{11} tags.

(D) Matrix analysis of pairwise classification accuracy across 96 cell populations expressing various FP_{11} tags, arranged by FP_{11} tag numbers shown in (A). ($n > 230$ cells per population).

(E) Analysis scheme for determining the maximum achievable minimum pairwise classification accuracy among randomly selected FP_{11}-tagged subpopulations.

(F) Graph showing the maximum achievable minimum pairwise classification accuracy among randomly selected FP_{11}-tagged subpopulations. For each subpopulation size, minimum accuracy between all possible pairs was determined from 10^7 random selections, with maximum values plotted.

(G) Comparative performance analysis of 30 candidate populations (detailed in Figures S3A and S3B) using both confocal microscopy (blue) and flow cytometry (orange). Analysis methodology follows that in (F). Data are presented as the mean ± SD from three independent experiments.

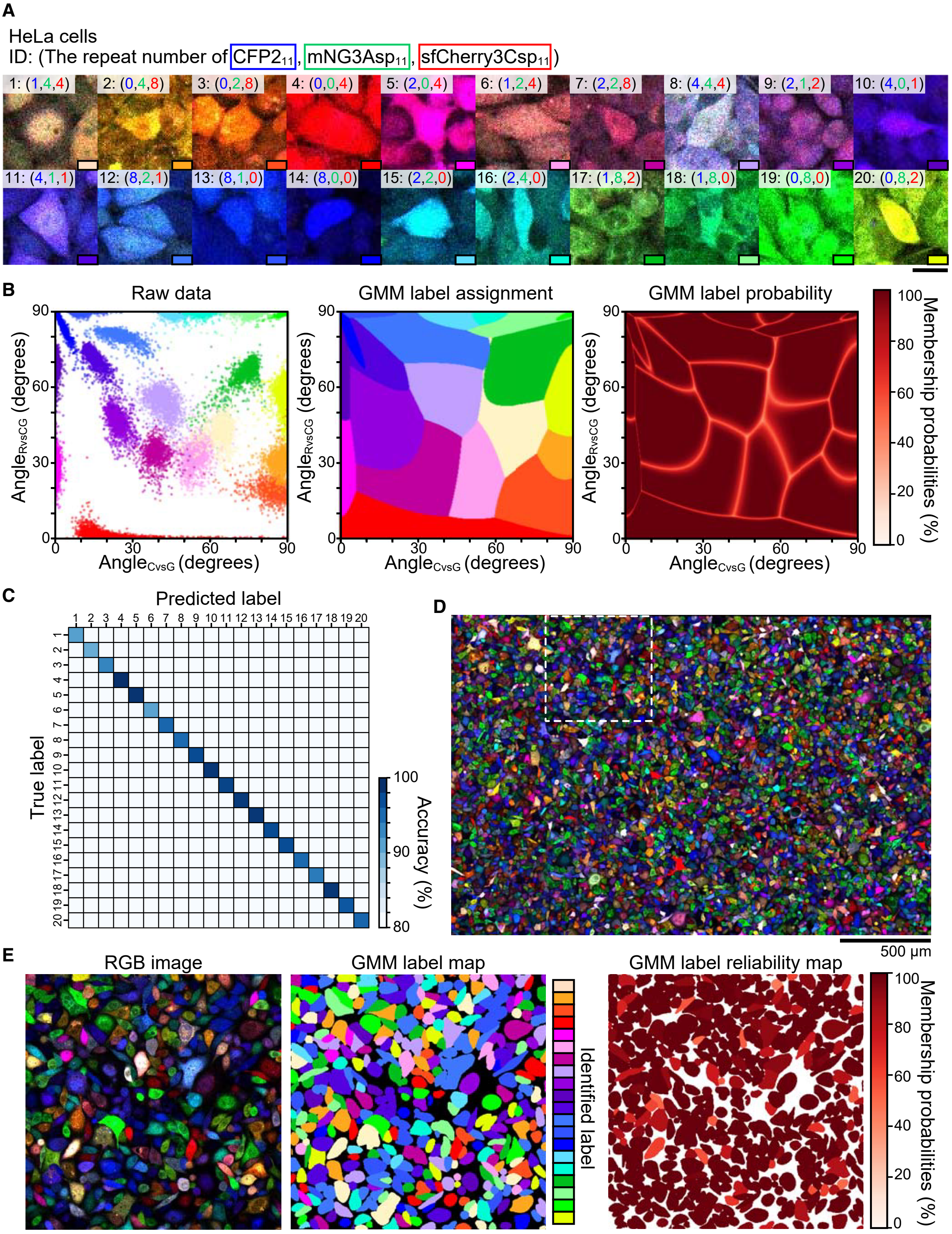

(legend on next page)

We conducted a comprehensive evaluation of the 30 candidate populations by using both confocal microscopy and flow cytometry, with three independent experimental replicates (Figures S3A and S3B). The relationship between population subset size and maximum achievable minimum pairwise classification accuracy was reassessed using these orthogonal detection methods (Figure 5G). While fluorescence microscopy demonstrated marginally superior classification accuracy, flow cytometry maintained robust discrimination capabilities, achieving >85% accuracy across a set of top 20 FP_{11} tags. The 2nd optimized set of 20 FP_{11} tags and their comprehensive pairwise discrimination analysis are presented in Figures S3C and S3D. Finally, we fine-tuned the copy numbers of single-color tags. We adopted $CFP2_{11}$(×8) and $mNG3Asp_{11}$(×8) to maximize the brightness. Meanwhile, we adopted $sfCherry3Csp_{11}$(×4) because $sfCherry3Csp_{11}$(×8) was found to aggregate at high expression levels (Figure S3E).

High-fidelity discrimination of 20 cell populations using optimized $FP_{1–10}$ expression platform

The $FP_{1–10}$ expression system was also refined by replacing the iRFP670 reporter with a puromycin resistance cassette. Through stringent puromycin selection, we established a HeLa clone cell ($HeLa^{FP1–10x3}$) expressing elevated levels of $CFP2_{1\text{-}10}$, $mNG3Asp_{1\text{-}10}$, and $sfCherry3Csp_{1\text{-}10}$. The optimized set of 20 FP_{11} tags was stably expressed in $HeLa^{FP1–10x3}$ cells, with individual cell populations analyzed by confocal microscopy. Fluorescence was visualized with distinct spectral channels: CFP2 (blue), mNG3Asp (green), and sfCherry3Csp (red). Merged images revealed unique signatures based on both color composition and intensity distribution (Figure 6A).

Cell segmentation was performed on maximum intensity projections of composite fluorescence signals, using Cellpose.[21] Mean fluorescence intensities were quantified across all three channels for each segmented cell. Following the methodology established in Figure 5C, we implemented spherical coordinate transformation to calculate $Angle_{CvsG}$ and $Angle_{RvsCG}$, generating a two-dimensional angular representation of relative fluorescence intensities (Figure 6B, left). These parameters were used to train a GMM, which established both label assignment distributions and label membership probability distributions in $Angle_{CvsG}$ and $Angle_{RvsCG}$ (Figure 6B, center and right). Validation of the trained GMM on independent test data demonstrated exceptional classification performance, achieving 97% accuracy in discriminating all 20 cell populations (Figure 6C). Using unsupervised *k*-means clustering on the 20 components helped achieve 96% accuracy in distinguishing between the 20 cell populations, even without requiring pre-training data for individual fluorescence profiles (Figure S4).

To evaluate system performance under practical conditions, we used pooled samples containing all 20 cell populations and acquired tiled images (Figure 6D). Application of the trained GMM enabled robust classification of individual cells, with 78% of cells being classified with more than 95% probability (Figure 6E).

Interaction dynamics in multi-population cell cultures of MDCK cells expressing EGF family ligands and their receptors

Finally, as an application of the developed labeling platform, we studied the contribution of heterotypic cell interactions mediated by epidermal growth factor (EGF) family ligands and their cognate receptors. For this, we used Madin-Darby Canine Kidney (MDCK) cells, which are widely used to study collective cell migration and cell competition regulated by the EGF signaling pathway. MDCK cells were labeled with the aforementioned $FP_{1–10}$ and 20 FP_{11} tags with 96% accuracy (Figures S5A and S5B). The labeled cells were co-cultured and time-lapse imaged to examine if we could accurately identify each cell population (Figure S5C; Video S1mmc2). By using a trained GMM, we classified 75% of cells with a probability greater than 95%. To validate classification accuracy, type 18 and type 20 cells labeled with the near-infrared nuclear dye DRAQ5 were each co-cultured with cells of the other 19 types (Figure S5D). Type 18 cells showed 95.2% sensitivity (true positive/ground truth), while type 20 cells showed 89.5% sensitivity. The GMM-labeled reliability values for misclassified cells were low (43.5–75.3), indicating a higher likelihood of incorrect labeling. Of note, the cell densities of 20 different labeled cell types at 72 h post-labeling showed only a 1.4-fold difference between the minimum and maximum values (Figure S5E), indicating that the expression of the fluorescent tags did not significantly affect the growth of MDCK cells.

Among the four EGF family ligands expressed in MDCK cells, we focused on heparin-binding EGF-like growth factor (HBEGF)

Figure 6. High-fidelity discrimination of 20 distinct cell populations using Caterpie

(A) Representative multicolor fluorescence micrographs of 20 distinct cell populations expressing unique FP_{11} tag combinations. Fluorescence channels: CFP2 (blue), mNG3Asp (green), and sfCherry3Csp (red). Copy numbers of each FP_{11} variant ($CFP2_{11}$, $mNG3Asp_{11}$, and $sfCherry3Csp_{11}$) are indicated in the upper left corner. Scale bars: 30 μm.

(B) Left: Scatterplot showing $Angle_{CvsG}$ versus $Angle_{RvsCG}$ distributions for 20 distinct cell populations from (A). $n > 1000$ cells per population. Center: Distribution of label assignments in $Angle_{CvsG}$ and $Angle_{RvsCG}$ based on Gaussian mixture model (GMM) classification. Right: Distribution of label membership probabilities in $Angle_{CvsG}$ and $Angle_{RvsCG}$.

(C) Classification performance matrix showing prediction accuracy against true population identities. Color intensity indicates classification accuracy. Overall average accuracy, 97%.

(D) Large-field composite image of pooled cell populations from (A), displaying CFP2 (blue), mNG3Asp (green), and sfCherry3Csp (red) fluorescence channels. Scale bars, 500 μm.

(E) Detailed analysis of region indicated by dashed box in (D). Left: Higher magnification of selected region. Center: Population assignment map following GMM-based classification into 20 distinct categories. Right: Visualization of GMM classification reliability through membership probability mapping.

and epiregulin (EREG). HBEGF, a high-affinity ligand, binds to heparan sulfate proteoglycans to provide strong signals restricted to short distances, promoting localized cell migration. In contrast, EREG, a low-affinity ligand, diffuses quickly and remotely, propagating signals approximately four times faster than high-affinity ligands and more efficiently to distant cells.[22] Both HBEGF and EREG bind to EGFR (ErbB1) and ErbB4. Using lentiviral transduction, we introduced EREG, HBEGF, EGFR (ErbB1), or ErbB4 in fluorescently labeled MDCK cells (Figure 7A). To investigate how EGF ligands and receptors with different affinities interact between heterotypic cells and generate spontaneous patterns, these four cell types, along with wild-type cells, were co-cultured for 8 days under 3% low-serum conditions. The resulting cell population was analyzed by fluorescence microscopy to identify each cell type (Figure 7B).

Before analyzing the positional relationships between heterotypic cells, we first examined the distribution patterns of homotypic cells, as cell types with limited motility and dispersal capability affect intercellular interactions with heterotypic cells. To examine homotypic cell clustering, each cell type is individually displayed (Figure 7C). Dispersion of each cell type was analyzed by the Clark-Evans index; values less than 1 indicate cellular clustering. We did not find significant homotypic cell clustering in any cell type (Figure 7D). We next analyzed the frequency of adjacent heterotypic cells. When examining receptor-expressing cells adjacent to EREG- or HBEGF-expressing cells (Figure 7E), EGFR-expressing cells showed significantly higher adjacency than wild-type or ErbB4-overexpressing cells, suggesting that EREG and HB-EGF serve as chemotactic factors to the EGFR-expressing cells. Furthermore, when comparing EREG and HBEGF, we found that EGFR- and ErbB4-expressing cells were more frequently adjacent to HBEGF-expressing cells than to EREG-expressing cells. These results indicate that EGFR receives ligand signals more efficiently than ErbB4 and that HBEGF provides a stronger proximal signaling effect than EREG.

In conclusion, the Caterpie method provides a versatile platform for analyzing cell populations that may exhibit homotypic and/or heterotypic clustering.

DISCUSSION

We have developed a cell identification platform that achieves robust identification of 20 distinct cell populations with 96%–97% accuracy and named this system "Cellular Array of Tunable Emission Reporter Proteins for Identification and Encoding" (Caterpie). Caterpie leverages fluorescence intensity modulation through engineered arrays of split FP fragments. The technological foundation of Caterpie comprises three complementary split FPs: split CFP2 and two newly engineered variants, namely split mNG3Asp and split sfCherry3Csp. To implement this system, we established a comprehensive toolkit consisting of 20 distinct FP_{11} tags, each constructed by fusing truncated SUMOstar protein to the 11th β-strand of the three split FPs in varying tandem repeat configurations. These engineered FP_{11} tags demonstrate highly specific labeling of $FP_{1–10}$-expressing cells, enabling population discrimination with 96%–97% accuracy through GMM analysis of the resulting multidimensional fluorescence signatures.

Current multicolor fluorescent labeling approaches can be broadly categorized into two distinct methodological frameworks. The first relies on stochastic recombination events mediated by site-specific recombinase systems such as Cre/*loxP* and Flp/FRT.[1,2,23–27] The second approach exploits the inherent randomness of transfection efficiencies or genomic integration sites.[28–32] While both established approaches depend on stochastic processes to generate fluorescent protein expression patterns, Caterpie represents a paradigm shift through its implementation of rationally designed color palettes. This deterministic approach enables precise targeting of specific cell populations and lineages. The system holds particular promise for applications requiring high-fidelity identification and longitudinal tracking of distinct cell types within complex biological contexts. However, the full realization of Caterpie's potential necessitates further optimization of delivery methodologies. Integration with the Landing Pad system,[33] which enables precise single-copy genomic integration, could further enhance this approach by providing controlled integration sites for multiplexed cellular labeling while maintaining the designed color palette integrity.

The theoretical prediction of an 8-fold signal enhancement for $FP_{11}(\times 8)$ constructs relative to their monomeric counterparts could not be fully realized in experimental measurements. Our studies with split CFP2 demonstrated approximately 5-fold enhancement in the median fluorescence intensity (Figure 2C). Further investigations revealed that the choice of fusion protein significantly impacts the signal amplification efficiency of octameric constructs (Figure S2C). In research using sfGFP, when ×3, ×4, or ×7 $sfGFP_{11}$ was fused to β-tubulin or lamin A/C, the length and brightness correlated well, nearly achieving theoretical limits.[4,6] However, when Teneurin-m was fused to ×7 $sfGFP_{11}$, only a 4-fold increase in brightness was achieved.[9] With the goal of reducing gene size, which is an advantage of our approach, we replaced the initially used histone H2B with ΔSUMOstar and achieved almost the same amplification rate as histone H2B, ×1.6 by each repetition (Figure S2E). This collective evidence suggests that achieving linear brightness amplification through multimerization of the 11th β-strand requires careful optimization of the fusion partner.

The application of computational protein structure prediction proved instrumental in enhancing split FP performance (Figure 3). This approach represents a significant departure from conventional optimization strategies, which typically rely on random mutagenesis.[34] Simultaneous introduction of mutations in both $\beta_{1\text{-}10}$ and β_{11} regions that enhance their mutual interactions proved challenging through random mutagenesis because the statistical probability of obtaining cooperative mutations is exceedingly low. Our successful implementation of structure prediction-guided optimization suggests a broader applicability of this methodology across other split FP systems.

In addition to these technical advances, our cellular analyses further highlight the functional significance of Caterpie in complex biological contexts. Around EREG- and HBEGF-overexpressing cells, the number of ErbB4-positive cells was comparable to that in wild-type cells, whereas EGFR-overexpressing cells were significantly enriched. This observation is consistent

CellPress
OPEN ACCESS

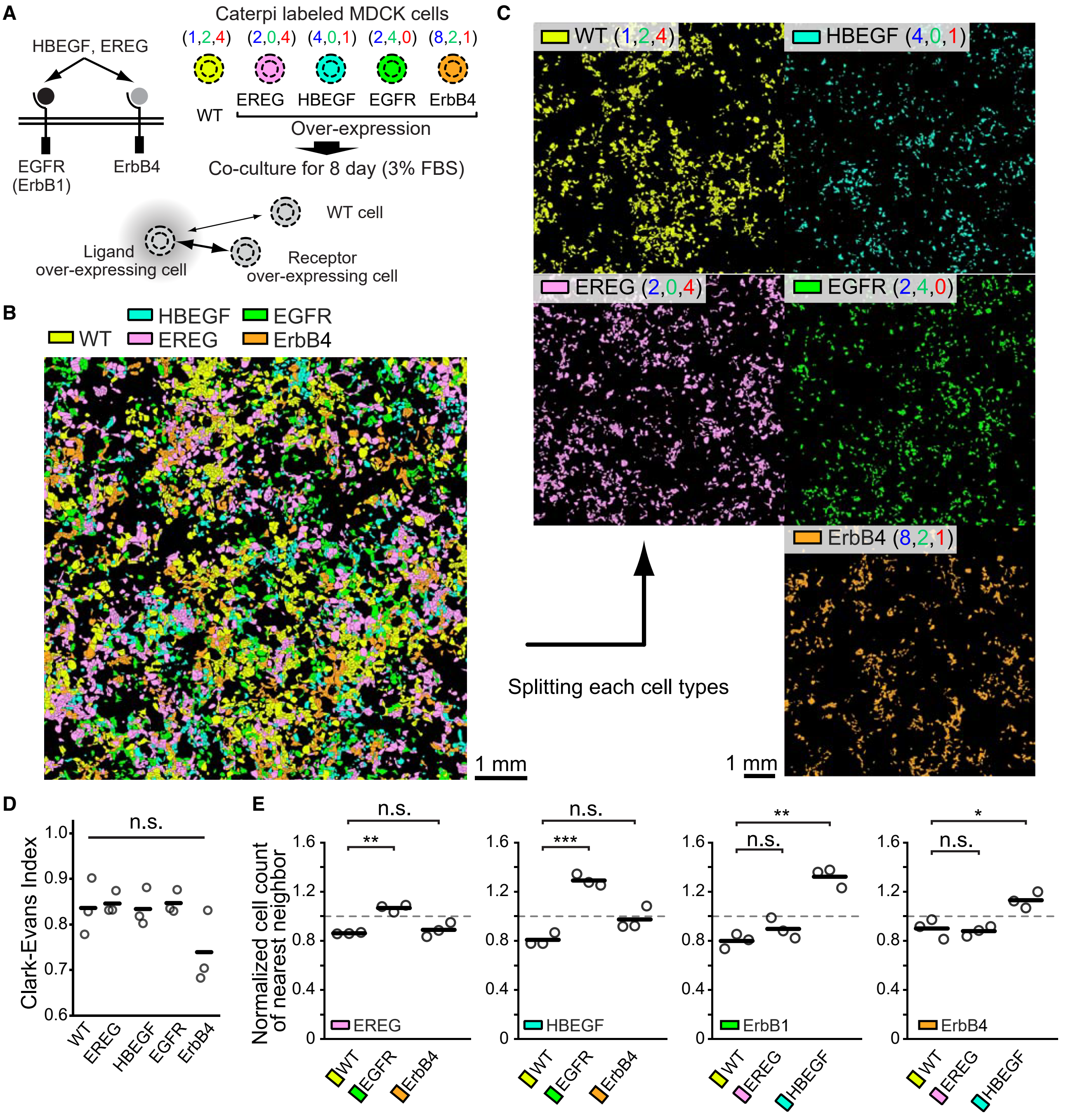

Figure 7. Interaction dynamics in multi-population cell cultures of MDCK cells expressing EGF family ligands and their receptors

(A) Schematic for the association pairs between EGF ligands (HBEGF and EREG) and EGF receptors (EGFR and ErbB4).

(B) Representative pseudocolored image of five distinct cell types cultured together and identified using the Caterpie method. Yellow, WT; Pink, EREG; Cyan, HBEGF; Green, EGFR; Orange, ErbB4. Copy numbers of each FP_{11} variant ($CFP2_{11}$, $mNG3Asp_{11}$, and $sfCherry3Csp_{11}$) are (1,2,4), (2,0,4), (4,0,1), (2,4,0), and (8,2,1), respectively. Scale bars: 1 mm.

(C) Individual display of the five cell types shown in (B). The re-expressed EGF signaling molecules and the copy numbers of FP_{11} tags are indicated in the upper left corner.

(D) Graph showing the Clark-Evans Index calculated for each of the five cell types. Data are from three independent experiments. Black lines represent the mean values across the three experiments. Statistical significance across the five groups was evaluated using a one-way ANOVA ($p = 0.1232$).

(E) Normalized nearest neighbor cell analysis for evaluating spatial relationships between cells. The ratio of heterotypic nearest neighbor counts observed in the actual data to those counted after randomly reassigning the labels of five cell types in the same dataset is shown. Data are from three independent experiments. Black lines represent the mean values across the three experiments.

with recent findings that EGFR, rather than other ErbB family members, serves as the principal receptor mediating ERK activity propagation through EGF signaling to regulate collective migration of MDCK cells.[35] Moreover, when comparing EREG and HBEGF, we found that EGFR- and ErbB4-positive cells were more frequently adjacent to HBEGF-expressing cells than to EREG-expressing cells, reflecting the strong short-range signaling elicited by HBEGF. Together, these results suggest that EGFR receives ligand signals more efficiently than ErbB4 and that the potent proximal signaling driven by HBEGF promotes the spatial clustering of EGFR-expressing cells to facilitate ERK activation and coordinated cell migration.

Limitations of the study

Importantly, Caterpie enables discrimination of these cellular subpopulations with high resolution. By exploiting fluorescence intensity information, rather than binary on/off states, Caterpie expands the number of distinguishable populations far beyond the conventional 2^n - 1 rule. For example, while three-color labeling yields only seven distinguishable populations (excluding unlabeled cells), Caterpie achieved discrimination of up to 20 populations, albeit with a modest decrease in accuracy to 96%–97%. This limitation was addressed by incorporating a reliability index for classification. Although Caterpie is currently limited to three compatible split FPs, the number of resolvable populations is expected to increase dramatically as additional fluorescent colors are engineered. At the same time, reliance on supervised training datasets becomes increasingly impractical as the number of populations expands. In this context, unsupervised clustering offers a promising alternative. Indeed, as demonstrated in Figure S4, even a relatively simple *k*-means clustering approach yielded 96% accuracy, suggesting that further improvements in identification precision will be achievable through careful selection of FP_{11} tags and refinement of clustering algorithms.

RESOURCE AVAILABILITY

Lead contact

Further information and requests for resources and reagents should be directed to and will be fulfilled by the lead contact, Michiyuki Matsuda (matsuda.michiyuki.87r@st.kyoto-u.ac.jp).

Materials availability

All plasmids generated in this study are available from the lead contact without restriction.

Data and code availability

- All data reported in this paper will be shared by the lead contact upon request. The source data for Figure 6 have been deposited at Figshare and is publicly available as of the date of publication at Figshare: https://doi.org/10.6084/m9.figshare.31351057.
- All original code has been deposited at Figshare and is publicly available at Figshare: https://doi.org/10.6084/m9.figshare.31351057 as of the date of publication.
- Any additional information required to reanalyze the data reported in this paper is available from the lead contact upon request.

ACKNOWLEDGMENTS

We are grateful to all members of the Matsuda Laboratory for their valuable input and discussions and to K. Hirano, M. Hirao, T. Uesugi, and K. Takakura for their excellent technical assistance. We thank Kotaro Tsuboyama and Yoshiho Ikeuchi, at the Institute of Industrial Science, The University of Tokyo, for providing experimental facilities and materials. This work was supported by the Kyoto University Live Imaging Center and the Komaba Analysis Core, Institute of Industrial Science, The University of Tokyo. Financial support was provided by JSPS KAKENHI grants (22KJ1998 to M.I.; 19H00993 and 20H05898 to M.M.; 23K23888, 24K21981, and 25H01362 to K.A.), a JST Moonshot R&D grant (JPMJMS2022 to M.M.), the Takeda Science Foundation (to K.A.), and an NIH grant (R35GM131923 to B.K.). M.I. was supported by a fellowship from the Japan Society for the Promotion of Science. T.K. was supported by fellowships from the Takeda Science Foundation, the Murata Overseas Scholarship Foundation, and the Japan Society for the Promotion of Science.

AUTHOR CONTRIBUTIONS

Conceptualization, M.I. and M.M.; resources, K.T., K.A., B.K., and M.M.; data curation, M.I., T.K., and M.M.; formal analysis, M.I., T.K., and Y.K.; supervision, M.M; funding acquisition, M.I., K.A., B.K., and M.M.; validation, M.I. and M.M.; investigation, M.I. and T.K.; visualization, M.I., T.K., and M.M.; methodology, M.I., T.K., B.K., and M.M.; writing – original draft, M.I. and T.K.; project administration, M.I. and M.M.; writing – review & editing, M.I., T.K., Y.K., K.T., K.A., B.K., and M.M.

DECLARATION OF INTERESTS

The authors declare no competing interests.

DECLARATION OF GENERATIVE AI AND AI-ASSISTED TECHNOLOGIES IN THE WRITING PROCESS

During the preparation of this work, the authors used Claude 4.5 Sonnet and ChatGPT-5 to improve the readability and language of the manuscript. After using these services, the authors reviewed and edited the content as needed and take full responsibility for the content of the publication.

STAR★METHODS

Detailed methods are provided in the online version of this paper and include the following:

- KEY RESOURCES TABLE
- EXPERIMENTAL MODEL AND STUDY PARTICIPANT DETAILS
  - Cell lines
- METHOD DETAILS
  - Plasmids
  - Vector construction for stable cell line generation
  - Establishment of cell lines
  - Fluorescence imaging
  - Fluorescence spectral analysis
  - Flow cytometry analysis
  - Lentivirus infection
  - Cell growth
- QUANTIFICATION AND STATISTICAL ANALYSIS
  - Computational design for enhanced β-barrel stability and split-strand complementation
  - Analysis procedure for cell identification using the Caterpie method
  - Cell classification using K-means
  - Clark-Evans index
  - Cell count of the nearest neighbor
  - Statistics

SUPPLEMENTAL INFORMATION

Supplemental information can be found online at https://doi.org/10.1016/j.crmeth.2026.101363.

CellPress
OPEN ACCESS

Received: July 28, 2025
Revised: November 8, 2025
Accepted: February 19, 2026
Published: March 26, 2026

REFERENCES

1. Livet, J., Weissman, T.A., Kang, H., Draft, R.W., Lu, J., Bennis, R.A., Sanes, J.R., and Lichtman, J.W. (2007). Transgenic strategies for combinatorial expression of fluorescent proteins in the nervous system. Nature *450*, 56–62.
2. Veling, M.W., Li, Y., Veling, M.T., Litts, C., Michki, S.N., Liu, H., Ye, B., and Cai, D. (2019). Identification of neuronal lineages in the Drosophila peripheral nervous system with a "digital" multi-spectral lineage tracing system. Cell Rep. *29*, 3303–3312.e3.
3. Cabantous, S., Terwilliger, T.C., and Waldo, G.S. (2005). Protein tagging and detection with engineered self-assembling fragments of green fluorescent protein. Nat. Biotechnol. *23*, 102–107.
4. Leonetti, M.D., Sekine, S., Kamiyama, D., Weissman, J.S., and Huang, B. (2016). A scalable strategy for high-throughput GFP tagging of endogenous human proteins. Proc. Natl. Acad. Sci. USA *113*, E3501–E3508.
5. Cho, N.H., Cheveralls, K.C., Brunner, A.D., Kim, K., Michaelis, A.C., Raghavan, P., Kobayashi, H., Savy, L., Li, J.Y., Canaj, H., et al. (2022). OpenCell: Endogenous tagging for the cartography of human cellular organization. Science *375*, eabi6983.
6. Kamiyama, D., Sekine, S., Barsi-Rhyne, B., Hu, J., Chen, B., Gilbert, L.A., Ishikawa, H., Leonetti, M.D., Marshall, W.F., Weissman, J.S., and Huang, B. (2016). Versatile protein tagging in cells with split fluorescent protein. Nat. Commun. *7*, 11046.
7. He, S., Cuentas-Condori, A., and Miller, D.M. (2019). Strategy for bright, tissue-specific GFP labeling of endogenous proteins in C. elegans. Genetics *212*, 387–395.
8. Goudeau, J., Sharp, C.S., Paw, J., Savy, L., Leonetti, M.D., York, A.G., Updike, D.L., Kenyon, C., and Ingaramo, M. (2021). Split-wrmScarlet and split-sfGFP: tools for faster, easier fluorescent labeling of endogenous proteins in Caenorhabditis elegans. Genetics *217*, iyab014.
9. Kamiyama, R., Banzai, K., Liu, P., Marar, A., Tamura, R., Jiang, F., Fitch, M.A., Xie, J., and Kamiyama, D. (2021). Cell-type-specific, multicolor labeling of endogenous proteins with split fluorescent protein tags in Drosophila. Proc. Natl. Acad. Sci. USA *118*, e2024690118.
10. Tamura, R., Jiang, F., Xie, J., and Kamiyama, D. (2021). Multiplexed labeling of cellular proteins with split fluorescent protein tags. Commun. Biol. *4*, 257.
11. Zhou, S., Feng, S., Brown, D., and Huang, B. (2020). Improved yellow-green split fluorescent proteins for protein labeling and signal amplification. PLoS One *15*, e0242592.
12. Feng, S., Varshney, A., Coto Villa, D., Modavi, C., Kohler, J., Farah, F., Zhou, S., Ali, N., Müller, J.D., Van Hoven, M.K., and Huang, B. (2019). Bright split red fluorescent proteins for the visualization of endogenous proteins and synapses. Commun. Biol. *2*, 344.
13. Kim, G., Lee, S., Levy Karin, E., Kim, H., Moriwaki, Y., Ovchinnikov, S., Steinegger, M., and Mirdita, M. (2025). Easy and accurate protein structure prediction using ColabFold. Nat. Protoc. *20*, 620–642.
14. Evans, R., O'Neill, M., Pritzel, A., Antropova, N., Senior, A., Green, T., Žídek, A., Bates, R., Blackwell, S., Yim, J., et al. (2022). Protein complex prediction with AlphaFold-Multimer. Preprint at bioRxiv. https://doi.org/10.1101/2021.10.04.463034.
15. Leaver-Fay, A., Tyka, M., Lewis, S.M., Lange, O.F., Thompson, J., Jacak, R., Kaufman, K., Renfrew, P.D., Smith, C.A., Sheffler, W., et al. (2011). ROSETTA3: An object-oriented software suite for the simulation and design of macromolecules. Methods Enzymol. *487*, 545–574.
16. Thieker, D.F., Maguire, J.B., Kudlacek, S.T., Leaver-Fay, A., Lyskov, S., and Kuhlman, B. (2022). Stabilizing proteins, simplified: A Rosetta-based webtool for predicting favorable mutations. Protein Sci. *31*, e4428.
17. Jumper, J., Evans, R., Pritzel, A., Green, T., Figurnov, M., Ronneberger, O., Tunyasuvunakool, K., Bates, R., Žídek, A., Potapenko, A., et al. (2021). Highly accurate protein structure prediction with AlphaFold. Nature *596*, 583–589.
18. Zhou, P., Lugovskoy, A.A., and Wagner, G. (2001). A solubility-enhancement tag (SET) for NMR studies of poorly behaving proteins. J. Biomol. NMR *20*, 11–14.
19. Liu, L., Spurrier, J., Butt, T.R., and Strickler, J.E. (2008). Enhanced protein expression in the baculovirus/insect cell system using engineered SUMO fusions. Protein Expr. Purif. *62*, 21–28.
20. Milech, N., Longville, B.A.C., Cunningham, P.T., Scobie, M.N., Bogdawa, H.M., Winslow, S., Anastasas, M., Connor, T., Ong, F., Stone, S.R., et al. (2015). GFP-complementation assay to detect functional CPP and protein delivery into living cells. Sci. Rep. *5*, 18329.
21. Stringer, C., Wang, T., Michaelos, M., and Pachitariu, M. (2021). Cellpose: a generalist algorithm for cellular segmentation. Nat. Methods *18*, 100–106.
22. Deguchi, E., Lin, S., Hirayama, D., Matsuda, K., Tanave, A., Sumiyama, K., Tsukiji, S., Otani, T., Furuse, M., Sorkin, A., et al. (2024). Low-affinity ligands of the epidermal growth factor receptor are long-range signal transmitters in collective cell migration of epithelial cells. Cell Rep. *43*, 114986.
23. Snippert, H.J., van der Flier, L.G., Sato, T., van Es, J.H., van den Born, M., Kroon-Veenboer, C., Barker, N., Klein, A.M., van Rheenen, J., Simons, B.D., and Clevers, H. (2010). Intestinal crypt homeostasis results from neutral competition between symmetrically dividing Lgr5 stem cells. Cell *143*, 134–144.
24. Tabansky, I., Lenarcic, A., Draft, R.W., Loulier, K., Keskin, D.B., Rosains, J., Rivera-Feliciano, J., Lichtman, J.W., Livet, J., Stern, J.N.H., et al. (2013). Developmental bias in cleavage-stage mouse blastomeres. Curr. Biol. *23*, 21–31.
25. Ghigo, C., Mondor, I., Jorquera, A., Nowak, J., Wienert, S., Zahner, S.P., Clausen, B.E., Luche, H., Malissen, B., Klauschen, F., and Bajénoff, M. (2013). Multicolor fate mapping of Langerhans cell homeostasis. J. Exp. Med. *210*, 1657–1664.
26. Cai, D., Cohen, K.B., Luo, T., Lichtman, J.W., and Sanes, J.R. (2013). Improved tools for the Brainbow toolbox. Nat. Methods *10*, 540–547.
27. Loulier, K., Barry, R., Mahou, P., Le Franc, Y., Supatto, W., Matho, K.S., Ieng, S., Fouquet, S., Dupin, E., Benosman, R., et al. (2014). Multiplex cell and lineage tracking with combinatorial labels. Neuron *81*, 505–520.
28. Weber, K., Bartsch, U., Stocking, C., and Fehse, B. (2008). A multicolor panel of novel lentiviral "gene ontology" (LeGO) vectors for functional gene analysis. Mol. Ther. *16*, 698–706.
29. García-Marqués, J., and López-Mascaraque, L. (2013). Clonal identity determines astrocyte cortical heterogeneity. Cereb. Cortex *23*, 1463–1472.
30. García-Moreno, F., Vasistha, N.A., Begbie, J., and Molnár, Z. (2014). CLoNe is a new method to target single progenitors and study their progeny in mouse and chick. Development *141*, 1589–1598.
31. Sakaguchi, R., Leiwe, M.N., and Imai, T. (2018). Bright multicolor labeling of neuronal circuits with fluorescent proteins and chemical tags. eLife *7*, e40350.
32. Kumamoto, T., Maurinot, F., Barry-Martinet, R., Vaslin, C., Vandormael-Pournin, S., Le, M., Lerat, M., Niculescu, D., Cohen-Tannoudji, M., Rebsam, A., et al. (2020). Direct readout of neural stem cell transgenesis with an integration-coupled gene expression switch. Neuron *107*, 617–630.e6.
33. Matreyek, K.A., Stephany, J.J., Chiasson, M.A., Hasle, N., and Fowler, D.M. (2020). An improved platform for functional assessment of large protein libraries in mammalian cells. Nucleic Acids Res. *48*, e1.
34. Feng, S., Sekine, S., Pessino, V., Li, H., Leonetti, M.D., and Huang, B. (2017). Improved split fluorescent proteins for endogenous protein labeling. Nat. Commun. *8*, 370.
35. Matsuda, K., Hirayama, D., Hino, N., Kuno, S., Sakaue-Sawano, A., Miyawaki, A., Matsuda, M., and Terai, K. (2023). Knockout of all ErbB-family

genes delineates their roles in proliferation, survival and migration. J. Cell Sci. *136*, jcs261199.

36. Yusa, K., Rad, R., Takeda, J., and Bradley, A. (2009). Generation of transgene-free induced pluripotent mouse stem cells by the piggyBac transposon. Nat. Methods *6*, 363–369.
37. Kawakami, K., Takeda, H., Kawakami, N., Kobayashi, M., Matsuda, N., and Mishina, M. (2004). A transposon-mediated gene trap approach identifies developmentally regulated genes in zebrafish. Dev. Cell *7*, 133–144.
38. Schindelin, J., Arganda-Carreras, I., Frise, E., Kaynig, V., Longair, M., Pietzsch, T., Preibisch, S., Rueden, C., Saalfeld, S., Schmid, B., et al. (2012). Fiji: an open-source platform for biological-image analysis. Nat. Methods *9*, 676–682.
39. Köker, T., Fernandez, A., and Pinaud, F. (2018). Characterization of split fluorescent protein variants and quantitative analyses of their self-assembly process. Sci. Rep. *8*, 5344.
40. Shcherbakova, D.M., and Verkhusha, V.V. (2013). Near-infrared fluorescent proteins for multicolor in vivo imaging. Nat. Methods *10*, 751–754.
41. Akagi, T., Sasai, K., and Hanafusa, H. (2003). Refractory nature of normal human diploid fibroblasts with respect to oncogene-mediated transformation. Proc. Natl. Acad. Sci. USA *100*, 13567–13572.
42. Komatsu, N., Terai, K., Imanishi, A., Kamioka, Y., Sumiyama, K., Jin, T., Okada, Y., Nagai, T., and Matsuda, M. (2018). A platform of BRET-FRET hybrid biosensors for optogenetics, chemical screening, and in vivo imaging. Sci. Rep. *8*, 8984.
43. Kim, J.H., Lee, S.R., Li, L.H., Park, H.J., Park, J.H., Lee, K.Y., Kim, M.K., Shin, B.A., and Choi, S.Y. (2011). High cleavage efficiency of a 2A peptide derived from porcine teschovirus-1 in human cell lines, zebrafish and mice. PLoS One *6*, e18556.
44. Kalderon, D., Roberts, B.L., Richardson, W.D., and Smith, A.E. (1984). A short amino acid sequence able to specify nuclear location. Cell *39*, 499–509.
45. Fischer, U., Huber, J., Boelens, W.C., Mattaj, I.W., and Lührmann, R. (1995). The HIV-1 Rev activation domain is a nuclear export signal that accesses an export pathway used by specific cellular RNAs. Cell *82*, 475–483.
46. Miyoshi, H., Blömer, U., Takahashi, M., Gage, F.H., and Verma, I.M. (1998). Development of a self-inactivating lentivirus vector. J. Virol. *72*, 8150–8157.
47. Stranges, P.B., and Kuhlman, B. (2013). A comparison of successful and failed protein interface designs highlights the challenges of designing buried hydrogen bonds. Protein Sci. *22*, 74–82.
48. Maguire, J.B., Haddox, H.K., Strickland, D., Halabiya, S.F., Coventry, B., Griffin, J.R., Pulavarti, S.V.S.R.K., Cummins, M., Thieker, D.F., Klavins, E., et al. (2021). Perturbing the energy landscape for improved packing during computational protein design. Proteins *89*, 436–449.
49. Pedregosa, F., Varoquaux, G., Gramfort, A., Michel, V., Thirion, B., Grisel, O., Blondel, M., Prettenhofer, R., Weiss, R., Dubourg, V., et al. (2011). Scikit-learn: Machine Learning in Python. J. Mach. Learn. Res. *12*, 2825–2830.

CellPress
OPEN ACCESS

STAR★METHODS

KEY RESOURCES TABLE

REAGENT or RESOURCE	SOURCE	IDENTIFIER
**Bacterial and virus strains**		
JM109 competent cell	SMO	Cat# CC0204
**Chemicals, peptides, and recombinant proteins**		
DMEM	Wako	Cat# 044-29765
FBS	Sigma-Aldrich	Cat# F7524
Penicillin-Streptomycin	Nacalai Tesque	Cat# 26253-84
DMEM/F-12, no phenol red	Gibco	Cat# 21041025
Cellmatrix Type I -C (Collagen, Type I, 3 mg mL-1, pH 3.0)	Nitta Gelatin	Cat# 637-00773
Puromycin dihydrochloride	Sigma-Aldrich	Cat# P-8833
Blasticidin S Hydrochloride	Wako	Cat# 029-18701
Hygromycin B	Wako	Cat# 31282-04-9
293fectin	Gibco	Cat# 12347019
DRAQ5	BioStatus Limited	Cat# DR50050
**Deposited data**		
Figure 6 Source Data 1	This paper	Figshare: https://doi.org/10.6084/m9.figshare.31351057
Figure 6 Source Data 2	This paper	Figshare: https://doi.org/10.6084/m9.figshare.31351057
Figure 6 Source Data 3	This paper	Figshare: https://doi.org/10.6084/m9.figshare.31351057
**Experimental models: Cell lines**		
Human: HeLa cells	Human Science Research Resources Bank	N/A
Human: Lenti-X 293T cells	Clontech	632180
Dog: MDCK cells	RIKEN BioResource Center	RCB0995
**Recombinant DNA**		
pCMV-mPBase(neo-)	Yusa et al.[36]	N/A
pCS-TP	Kawakami et al.[37]	N/A
pCMV-VSVG-RSV-Rev	A gift from Hiroyuki Miyoshi (RIKEN BioResource Center, Japan)	N/A
psPAX2	A gift from Didier Trono (Ecole Polytechnique Fé dérale de Lausanne, Switzerland)	Addgene plasmid #12260
**Software and algorithms**		
ColabFold	Kim et al.[13]	RRID: SCR_025453
Rosetta	Leaver-Fay et al.[15]	RRID: SCR_015701
Fiji	Schindelin et al.[38]	RRID:SCR_002285
MATLAB	MathWorks	RRID:SCR_001622
Python	Python Software Foundation	RRID:SCR_008394
Cellpose	Stringer et al.[21]	RRID: SCR_021716
RosettaScripts protocol for site-saturation mutagenesis	This paper	Figshare: https://doi.org/10.6084/m9.figshare.31351057
RosettaScripts protocol for point mutation and mutation cluster interface design	This paper	Figshare: https://doi.org/10.6084/m9.figshare.31351057
Python program for Caterpie cell classification using Gaussian Mixture Models	This paper	Figshare: https://doi.org/10.6084/m9.figshare.31351057

EXPERIMENTAL MODEL AND STUDY PARTICIPANT DETAILS

Cell lines

HeLa cells and Lenti-X 293T cells were obtained from the Human Science Research Resources Bank and Clontech, respectively. Both cell lines were maintained in DMEM (Wako Pure Chemical Industries) supplemented with 10% fetal bovine serum (Sigma-Aldrich) and 1% penicillin/streptomycin (Nacalai Tesque). MDCK (ECACC 84121903) cells were purchased from the European Collection of Authenticated Cell Cultures (ECACC) through the RIKEN BioResource Center (no. RCB0995) and maintained in DMEM (Wako Pure Chemical Industries) supplemented with 10% fetal bovine serum (Sigma-Aldrich), 1% penicillin/streptomycin (Nacalai Tesque).

METHOD DETAILS

Plasmids

The following cDNAs were synthesized with optimized codons by GeneArt (Thermo Fisher Scientific, Waltham, MA): sfGFP$_{1-10}$,[3] CFP2$_{1-10}$,[39] mNeonGreen3A$_{1-10}$,[11] sfCherry3Csp$_{1-10}$, mRuby4$_{1-10}$,[10] GB1,[18] ΔSUMOstar.[19] Additional split fluorescent protein variants were generated through site-directed mutagenesis of existing templates: Cerulean$_{1-10}$[10] and YFP1$_{1-10}$[39] from sfGFP$_{1-10}$, mNeonGreen3K$_{1-10}$[11] from mNeonGreen3A$_{1-10}$, and sfCherry3C$_{1-10}$[12] from sfCherry2$_{1-10}$ (Addgene: #82603). The Histone H2B coding sequence was subcloned from Addgene (plasmid # 11680 for Histone H2B; Cambridge, MA).

Vector construction for stable cell line generation

A base Tol2 transposon vector (pT2A-IRES-iRFP670) was constructed by subcloning iRFP670 cDNA[40] with an internal ribosome entry site (IRES)[41] into the pT2A vector.[37] CFP2$_{1-10}$ cDNA was subsequently PCR-amplified and inserted into this base vector using In-Fusion assembly (Clontech, Mountain View, CA) to generate pT2A_CFP2$_{1-10}$-IRES-iRFP670. Two distinct vector backbones, pT2A_IRES-iRFP670 and pT2ADW (containing IRES-puro cassette),[42] were used for multicistronic construct assembly. The following elements were PCR-amplified and assembled into both vectors using In-Fusion: sfCherry3Csp$_{1-10}$, mNeonGreen3Asp$_{1-10}$ with self-cleaving P2A peptide,[43] and CFP2$_{1-10}$ with P2A peptide. Stable cell lines were generated through co-transfection of the constructed pT2A vectors with pCS-TP (obtained from Kawakami et al., 2004[37]).

To generate pPBpuro_EF1a constructs containing CFP2$_{11}$(×1), CFP2$_{11}$(×2), CFP2$_{11}$(×4), CFP2$_{11}$(×8) -mCherry-NLS, cDNAs encoding Histone H2B, self-cleaving peptide P2A sequences, mCherry, and the nuclear localization signal (NLS) of the SV40 large T antigen (PKKKRKV)[44] were PCR-amplified and assembled using In-Fusion into pPBpuro_EF1a vectors (a kind gift from K. Yusa), yielding pPBpuro_EF1a_Histone H2B-P2A-mCherry-NLS. Either synthesized CFP2$_{11}$ or CFP2$_{\text{Full-length}}$ cDNA (created by PCR amplification of CFP2$_{1-10}$ cDNA and CFP2$_{11}$) was then assembled into pPBpuro_EF1a_Histone H2B-P2A-mCherry-NLS, resulting in pPBpuro_EF1a_CFP2$_{\text{Full-length}}$/CFP2$_{11}$(×1)-mCherry-NLS.

Because long repetitive nucleotide sequences cannot be directly synthesized in current DNA synthesis technology, we adopted concatemerization approach to construct tandem repeats. Restriction enzyme sites, BglII, BamHI/NotI, were introduced before and after FP$_{11}$, respectively. To generate CFP2$_{11}$(×2), the CFP2$_{11}$(×1) insert and vector were digested with BglII/NotI and BamHI/NotI, respectively, and then ligated. Subsequently, CFP2$_{11}$(×4) and CFP2$_{11}$(×8) were generated using the same approach. By substituting the CFP2$_{11}$ insert with sfCherry3Csp$_{11}$ or mNeonGreen3Asp$_{11}$ inserts, multiple tandem repeats of these fragments were constructed using the same strategy. Various modifications were made to customize the constructs for specific experimental requirements: the puromycin-resistance gene (puro) was replaced with the blasticidin S-resistance gene (bsr); Histone H2B was substituted with GB1, ΔSUMOstar; mCherry was replaced with EBFP2 or mTagBFP2; and the NLS was substituted with the nuclear export signal (NES) of the HIV-1 rev protein (LPPLERLTLD).[45]

Mutant variants of sfCherry3C$_{1-10}$ were generated using overlap extension PCR and assembled into pPB-based vectors[36] containing IRES-bsr (blasticidin S-resistance gene) using In-Fusion. Additional mutant variants of mNeonGreen3A$_{11}$ and sfCherry3C$_{11}$ were synthesized and inserted into pPB-based vectors[36] containing IRES-puro (puromycin-resistance gene) using Ligation High Ver. 2 (TOYOBO). Stable cell lines were established through co-transfection of these constructs with pCMV-mPBase (obtained from the Wellcome Trust Sanger Institute).

Establishment of cell lines

For transposon-mediated gene transfer, pT2A_CFP2$_{1-10}$-IRES-iRFP670, or pT2A_FP$_{1-10}$x3-IRES-iRFP670 was cotransfected with pCS-TP into HeLa cells by using 293fectin (Thermo Fisher Scientific, Waltham, MA). These obtained HeLa cells were sorted using an FACS Aria IIIu cell sorter (Becton Dickinson, Franklin Lakes, NJ) based on iRFP670 fluorescence to achieve a high expression level of the CFP2$_{1-10}$ or FP$_{1-10}$ × 3. Single-cell cloning of these sorted populations to yield HeLa$^{\text{CFP2 1-10_iRFP670}}$ cells and HeLa$^{\text{FP1-10x3_iRFP670}}$ cells.

pT2ADW_FP$_{1-10}$ × 3 (containing IRES-puro cassette) was cotransfected with pCS-TP using either 293fectin (Thermo Fisher Scientific, Waltham, MA) for HeLa cells or electroporation with an Amaxa nucleofector (Lonza, Basel, Switzerland) for MDCK cells. The transfected cells were selected with 5 μg mL^{-1} puromycin (no. P-8833; Sigma-Aldrich), followed by single-cell cloning to yield HeLa$^{\text{FP1-10x3}}$ cells or MDCK$^{\text{FP1-10x3}}$ cell lines.

CellPress
OPEN ACCESS

Fluorescence imaging

For evaluation of split fluorescent proteins, HeLa cells in 24-well plates were co-transfected with 100 ng of FP_{11} plasmid [$FP_{Full\text{-}length}$, $FP_{11}(\times 1)$, or $FP_{11}(\times 4)$] and 400 ng of $FP_{1\text{-}10}$ plasmid using 293fectin (Thermo Fisher Scientific, Waltham, MA). Cells were seeded onto collagen type I-coated (Nitta Gelatin, Osaka, Japan) glass-based 24-well plates (AGC Inc., Tokyo, Japan) and cultured for 48 h. Prior to imaging, cells were equilibrated for at least 1 h in DMEM/F-12, no phenol red (Gibco) supplemented with 10% fetal bovine serum (Sigma-Aldrich), and penicillin/streptomycin (Nacalai Tesque).

For FP_{11} labeling, pPBbsr_EF1a-ΔSUMOstar-$CFP2_{11}$(xa)-$mNeonGreen3Asp_{11}$(xb)-$sfCherry3Csp_{11}$(xc) was cotransfected with pCMV-mPBase into $HeLa^{FP1\text{-}10x3}$ cells by using 293fectin or $MDCK^{FP1\text{-}10x3}$ cells using electroporation. Transfected cells were selected with 10 μg mL^{-1} blasticidin S (Wako) and seeded onto collagen type I-coated glass-based plates.

Cells were observed with a Leica TCS SP8 FALCON confocal microscope (Leica-Microsystems, Wetzlar, Germany) equipped with an HC PL APO 20×/0.75 dry CS2 objective, an HC PL APO 40×/1.30 OIL CS2 objective, Leica HyD SMD detectors, a white light laser of 80 MHz pulse frequency, a Diode 405 (VLK 0550 T01; LASOS, Jena, Germany), a 440 nm diode laser (PDL 800-D; PicoQuant, Berlin, Germany), and a stage top incubator (Tokai Hit, Fujinomiya, Japan) to maintain 37°C and 5% CO2. The following excitation wavelengths and emission band paths were used for the imaging: for EBFP2 imaging, 405 nm excitation, 410–450 nm emission; Cerulean or CFP2 imaging, 440 nm excitation, 460–490 nm emission; for sfGFP imaging, 488 nm excitation, 500–550 nm emission; for YFP1 imaging, 514 nm excitation, 520–550 nm emission; for mNG3A, mNG3K, or mNG3Asp imaging, 506 nm excitation, 510–550 nm emission; for sfCherry3C or sfCherry3Csp imaging, 594 nm excitation, 600–645 nm emission; for mRuby4 imaging, 561 nm excitation, 580–645 nm emission; for iRFP670 imaging, 650 nm excitation, 660–760 nm emission. To eliminate the background signal, the time gate for fluorescence detection was set from 1.0 ns to 6.0 ns.

In the multiplex imaging, the following excitation wavelengths and emission band paths were used: for CFP2 imaging, 440 nm excitation, 450–500 nm emission; for mNG3Asp imaging, 506 nm excitation, 515–580 nm emission; for sfCherry3Csp imaging, 594 nm excitation, 605–645 nm emission. To eliminate the background signal, the time gate for mNG3Asp and sfCherry3Csp fluorescence detection was set from 0.2 ns to 6.0 ns.

Images were processed and analyzed with FIJI.[38] For quantification of fluorescent intensity, images were segmented using Cellpose algorithm.[21] Fluorescent intensity calculation was performed using custom MATLAB (MathWorks) and Python scripts.

Fluorescence spectral analysis

Fluorescence spectra were measured by fluorescence microscopy. Images were acquired with an HC PL APO 20×/0.75 dry CS2 objective using excitation at either 470 nm for split mNG3A or 546 nm for split sfCherry3C.

Flow cytometry analysis

Cells were suspended in PBS containing 3% FBS and analyzed or sorted with a FACS Aria IIIu cell sorter (Becton Dickinson, Franklin Lakes, NJ). The following laser and emission filter combinations were used for fluorescence detection: CFP2, a 405 nm laser and an ET470/24m filter (Chroma Technology Corp., Bellows Falls, VT); mNeonGreen3Asp, a 488 nm laser, and a DF530/30 filter; sfCherry3Csp, a 561 nm laser and a DF582/15 filter (Omega Optical); iRFP670, a 633 nm laser and a DF660/20 filter (Omega Optical). Cell debris and aggregates were excluded by gating for size and granularity. Laser area scaling factors were adjusted to optimize signal linearity as follows: violet laser, 0.49; blue laser, 0.40; yellow-green laser, 0.43; and red laser, 0.44. The FSC area scaling factor was set to 0.38. Photomultiplier tube (PMT) voltages were set as follows: FSC, 250; SSC, 390; 405–470 channel, 520; 488–530 channel, 400; 561–582 channel, 500; and 633–660 channel, 569. In addition to doublet exclusion, cells exhibiting positive iRFP670 fluorescence were selected, and only these cells were used for further analysis. Data analysis was performed using FlowJo software (Tree Star, Ashland, OR). For background correction, the median fluorescence intensities of CFP2, mNeonGreen3Asp, and sfCherry3Csp after gating were measured in $HeLa^{FP1\text{-}10\times3\_iRFP670}$ cells lacking FP_{11} tags, and these values were subtracted from the corresponding fluorescence intensities of each experimental sample. The classification program used for flow cytometry data was identical to that used for microscopy-based analysis. Details of the classification procedure are described in "analysis procedure for cell identification using the Caterpie method."

Lentivirus infection

For lentivirus production, the EGF ligand or receptor expressing plasmid, psPAX2 (Addgene no. 12260), and pCMV-VSV-G-RSV-Rev[46] were co-transfected into Lenti-X 293T cells using polyethylenimine (no. 24765-1; Polyscience Inc.). The infected cells were selected with media containing the following antibiotics, depending on the drug resistance genes carried by the EGF ligand or receptor expressing plasmids: 200 μg mL^{-1} hygromycin (no. 31282-04-9; Wako).

Cell growth

For quantifying cell growth, MDCK cells labeled by Caterpie were seeded on collagen-coated 24-well glass-bottom plates (AGC Inc., Tokyo, Japan) at a density of 5×10^4 cells/mL. After 1 h of incubation, the medium was replaced with DMEM/F-12, no phenol red supplemented with 100 units/mL penicillin, 100 μg/mL streptomycin, and 10% FBS. Following cell seeding, cells were observed using a confocal microscope (Leica SP8) every 24 h for 3 days. Observation conditions are written in the "fluorescence imaging" section. Cell numbers were counted using the Cellpose segmentation algorithm.

QUANTIFICATION AND STATISTICAL ANALYSIS

Computational design for enhanced β-barrel stability and split-strand complementation

The tertiary structures of split mNG3Asp and split sfCherry3Csp were modeled using ColabFold[13] implementation of AlphaFold Multimer[14] or AlphaFold2[17] and visualized with PyMOL (http://www.pymol.org/). The highest-confidence model of the split sfCherry3Csp was then adopted as the starting coordinate for downstream Rosetta-based design.

To enhance global β-barrel stability of split sfCherry3Csp, *in silico* site-saturation mutagenesis (SSM) of all 224 positions in sfCherry3C$_{1\text{-}10}$ and sfCherry3C$_{11}$ was performed with a Rosetta-based workflow adapted from Thieker et al.[16] For each variant, side-chain and backbone sampling were confined to a 10 Å sphere around the mutated residue, while the remainder of the protein was held fixed under coordinate constraints (sd = 1 outside the design sphere; sd = 2 within a surrounding "soft sphere"). Each mutant underwent three cycles of Cartesian FastRelax (MonomerRelax2019), and the change in Rosetta energy (ΔE) was calculated as the difference in total_score between each mutant and the wild-type model using the ref. 2015 score function.

To reinforce the $\beta_{1\text{-}10}/\beta_{11}$ interface, two design workflows, Point-Mutation (PM) and Mutation Cluster (MC), were adapted from the workflow of Thieker et al.[16] and modified for interface optimization. (1) In the PM workflow, SSM of the β_{11} fragment (residues 1–16) was carried out, with the addition of an InterfaceAnalyzer[47] to compute binding energy (dG_separated) and packing statistics. Residues within 10 Å of each mutation site were sampled under the same constraint scheme used for stability design, while non-neighbor residues were held fixed; each point mutant was relaxed by Cartesian FastRelax prior to interface analysis, and final dG_separated values were calculated using the unconstrained ref. 2015 Rosetta score function. (2) In the MC workflow, the seed mutations from the PM step were used as input for a FastDesign protocol[48] using the InterfaceDesign2019 relax script to generate clustered mutations. The neighbor-selection radius was extended to 12 Å, and an inner design shell was defined by residues both in direct atomic contact (≤5–7 Å, via a CloseContact selector) and satisfying the InterfaceByVector geometric filter. A ResidueTypeConstraintGenerator was applied to inner-shell, biasing retention of wild-type identities. Critical chromophore-interacting residues were protected by marking them non-designable. Multi-residue combinations sampled via FastDesign were evaluated with InterfaceAnalyzer for dG_separated and packing quality. The scores were obtained under the unconstrained ref. 2015 energy function.

Analysis procedure for cell identification using the Caterpie method

Processing of fluorescence intensity data

Three-dimensional fluorescence intensity data were acquired from individual cells by measuring CFP2, mNG3Asp, and sfCherry3Csp signals using either fluorescence microscopy or flow cytometry, followed by subtraction of background fluorescence obtained from non-fluorescent control cells. Data obtained from both modalities were analyzed using the same classification framework. Prior to angular calculations, fluorescence intensities of CFP2, mNG3Asp, and sfCherry3Csp were normalized by dividing each value by the global median intensity of the corresponding fluorophore, calculated across all 20 cell types combined. The normalized intensities were then represented in a three-dimensional Cartesian coordinate system, with CFP2 on the x axis, mNG3Asp on the y axis, and sfCherry3Csp on the z axis. For each cell, Cartesian coordinates (x, y, z) were transformed into spherical coordinates (r, $\text{Angle}_{\text{CvsG}}$, $\text{Angle}_{\text{RvsCG}}$) using a custom Python script, according to the following equations.

$$r = (x^2 + y^2 + z^2)^{1/2}$$

$$\text{Angle}_{\text{CvsG}} = \tan^{-1}(y/x)$$

$$\text{Angle}_{\text{RvsCG}} = \cos^{-1}(z/r)$$

Training of the Caterpie classification model

The classification model was trained using Gaussian Mixture Models (GMMs) implemented in the scikit-learn library.[49] A single Gaussian distribution was first fitted to a training subset of each of the 20 cell populations, and the resulting class-specific means and covariance matrices were concatenated to construct the GMM. The training dataset comprised 80% of the total data ($\sim 2.9 \times 10^4$ cells, with $>10^3$ cells per population), using the two angular features (AngleCvsG and AngleRvsCG). The remaining 20% of the data served as the test set for classification. Predictions from the trained GMM were compared with the ground truth to evaluate model accuracy, which was visualized using a confusion matrix (Figure 6C).

Classification of mixed cell populations

The trained GMM was then applied to angular data calculated from the fluorescence intensities of pooled cells, and the predicted cell labels were visualized. For each class k, we calculated the probability of each input data point belonging to the corresponding GMM model, representing the likelihood of class membership:

$$P(x|\theta_k) = N(x|\mu_k, \Sigma_k)$$

where x is the input data point, θ_k represents the parameters of the k-th GMM model, and $N(x|\mu_k, \Sigma_k)$ denotes the normal distribution with mean μ_k and covariance Σ_k. Subsequently, the probabilities were normalized such that the sum of probabilities across all classes equals 1 for each data point:

$$P(k|x) = P(x|\theta_k) \ / \ \Sigma_j \ P(x|\theta_j)$$

We visualized the membership probabilities of the predicted classes. All computational analyses were performed using custom-made Python scripts. By using three sample datasets—fluorescence intensities of 20 training samples, fluorescence intensities of pooled cells, and positions of pooled cells—the identification program can be tested.

Cell classification using K-means

We used $Angle_{CvsG}$ and $Angle_{RvsCG}$ as the feature space coordinates for clustering. K-means clustering was performed on the resulting dataset using scikit-learn's KMeans algorithm with 21 clusters (random_state = 0). As shown in Figure 6B, cells labeled with tag (B,G,R)=(0,0,4) corresponding to ID #4 exhibited a wide distribution of values in the $Angle_{CvsG}$-$Angle_{RvsCG}$ space, resulting in their identification as two distinct clusters that were subsequently merged. This accounts for the selection of 21 clusters in our analysis. Classification accuracy was assessed by comparing the remapped cluster assignments with ground truth labels and visualized using a confusion matrix. The overall classification accuracy was calculated as the percentage of correctly classified cells. All calculations were performed using Python.

Clark-Evans index

We analyzed the spatial distribution patterns of five cell types. Individual cells were first segmented using Cellpose, with centroids calculated and classified into five cell types via the Caterpie method. For spatial analysis, we extracted the coordinates of each cell type, calculated nearest neighbor distances using a k-dimensional tree algorithm, and computed the Clark-Evans index (R). This index compares observed mean distances to those expected under complete spatial randomness, where $R = 1$ indicates random distribution, $R < 1$ indicates clustering, and $R > 1$ indicates regularity. All calculations were performed using Python.

Cell count of the nearest neighbor

We analyzed the spatial relationships between different cell types using a nearest-neighbor approach. Cells were classified into five distinct populations based on fluorescent labels. For each cell of the target population (label 1), we calculated the distance to every cell of other populations (labels 2–5) and identified the closest neighboring cell using a k-dimensional tree algorithm. To assess the statistical significance of the observed patterns, we generated 100 randomized distributions for each dataset by shuffling cell positions while preserving the total number of each cell type. To determine whether the observed spatial associations between cell types differed significantly from random expectations, we calculated the ratio of the frequency with which each non-target cell type appeared as the nearest neighbor to target cells in the original distribution divided by the mean frequency observed across 100 randomized distributions. All calculations were performed using Python.

Statistics

All statistical analyses were carried out using Python. No statistical analysis was used to predetermine the sample size. An unpaired Welch's t test was used for pairwise comparisons unless otherwise stated. For Figure 7D, a one-way ANOVA was performed to compare means across five groups. Data are expressed as the mean ± s.d. p-values of less than 0.05 were considered to be statistically significant in two-tailed tests and were classified into four categories: $*p < 0.05$, $**p < 0.01$, $***p < 0.001$, and n.s. (not significant, i.e., $p \geq 0.05$).

Cell Reports Methods, Volume *6*

Supplemental information

Intensity modulation of trichromatic split

fluorescent proteins for live cell mapping

Mamoru Ishii, Tomoaki Kinjo, Yohei Kondo, Kenta Terai, Kazuhiro Aoki, Brian Kuhlman, and Michiyuki Matsuda

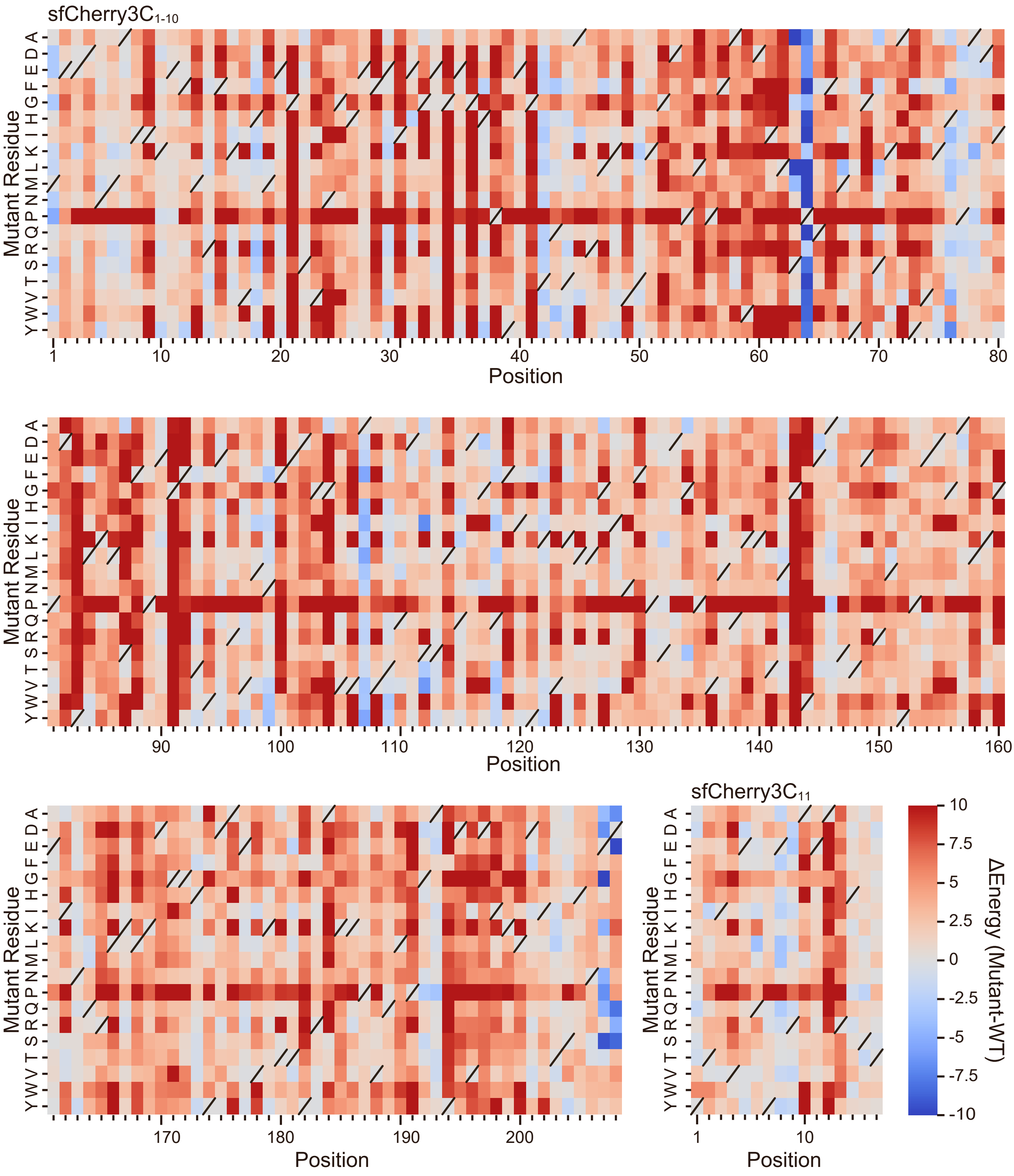

Figure S1: Comprehensive mutational scanning of split sfCherry3C, related Figure 3.

Heatmap displaying the ΔEnergy values for saturating mutations in both $sfCherry_{1\text{-}10}$ and $sfCherry_{11}$. Black slashes indicate wild-type amino acids.

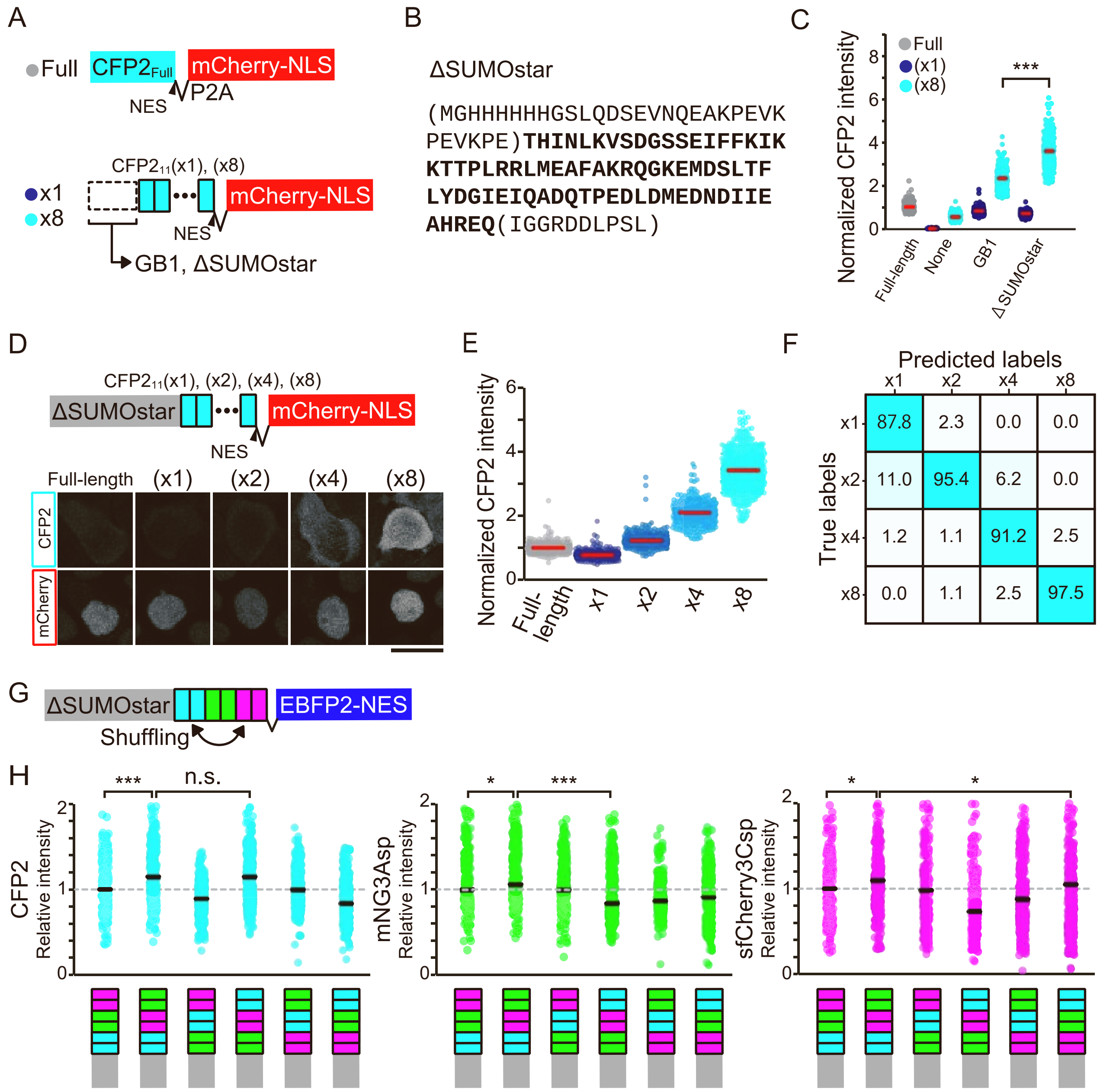

	x1	x2	x4	x8
x1	87.8	2.3	0.0	0.0
x2	11.0	95.4	6.2	0.0
x4	1.2	1.1	91.2	2.5
x8	0.0	1.1	2.5	97.5

Figure S2: Optimization of the FP$_{11}$ tag, related to Figure 5.

(A) Schematic representation of expression constructs: CFP2$_{Full\text{-}length}$-NES and CFP2$_{11}$ variants [(x1) or (x8)]-NES with or without GB1 or ΔSUMOstar (truncated SUMOstar) tags.
(B) Amino acid sequences of SUMOstar, with ΔSUMOstar region highlighted in bold.
(C) Bee swarm plots showing quantitative analysis of normalized CFP2 fluorescence in HeLa cells co-expressing CFP2$_{1\text{-}10}$ with either CFP2$_{Full\text{-}length}$-NES or CFP2$_{11}$(x1)/CFP2$_{11}$(x8)-NES variants, with or without GB1 or ΔSUMOstar tags (constructs shown in panel A). Median values are indicated by red lines.
(D) Construct architecture and expression analysis: Upper panel shows schematics of ΔSUMOstar fusions containing varying copy numbers of CFP2$_{11}$ [CFP2$_{11}$(x1), (x2), (x4), and (x8)]. Lower panel presents representative confocal micrographs of HeLa cells stably co-expressing CFP2$_{1\text{-}10}$ with either CFP2$_{Full\text{-}length}$-NES or ΔSUMOstar-tagged CFP2$_{11}$ variant. Scale bar: 20 µm.
(E) Comparative analysis of normalized CFP2 fluorescence in HeLa cells expressing ΔSUMOstar-tagged CFP2$_{11}$ variants [(x1), (x2), (x4), or (x8)] shown in panel D. Data presented as bee swarm plots with median values indicated by red lines.
(F) Classification performance matrix showing predictive accuracy against true population identities (rows), with color intensity indicating classification accuracy.
(G) Schematic representation of expression constructs: ΔSUMO-tagged FP$_{11}$ arrays comprising paired repeats of CFP2$_{11}$, mNG3Asp$_{11}$, and sfCherry3Csp$_{11}$ [(x2) each, total (x6)].
(H) Quantitative analysis presented as bee swarm plots showing normalized fluorescence intensities (CFP2, mNG3Asp, and sfCherry3Csp) in HeLa cells expressing six different permutations of FP$_{11}$ repeat arrangements, with concurrent FP$_{1\text{-}10}$x3 expression. Data obtained by confocal microscopy, with median values indicated by black lines.

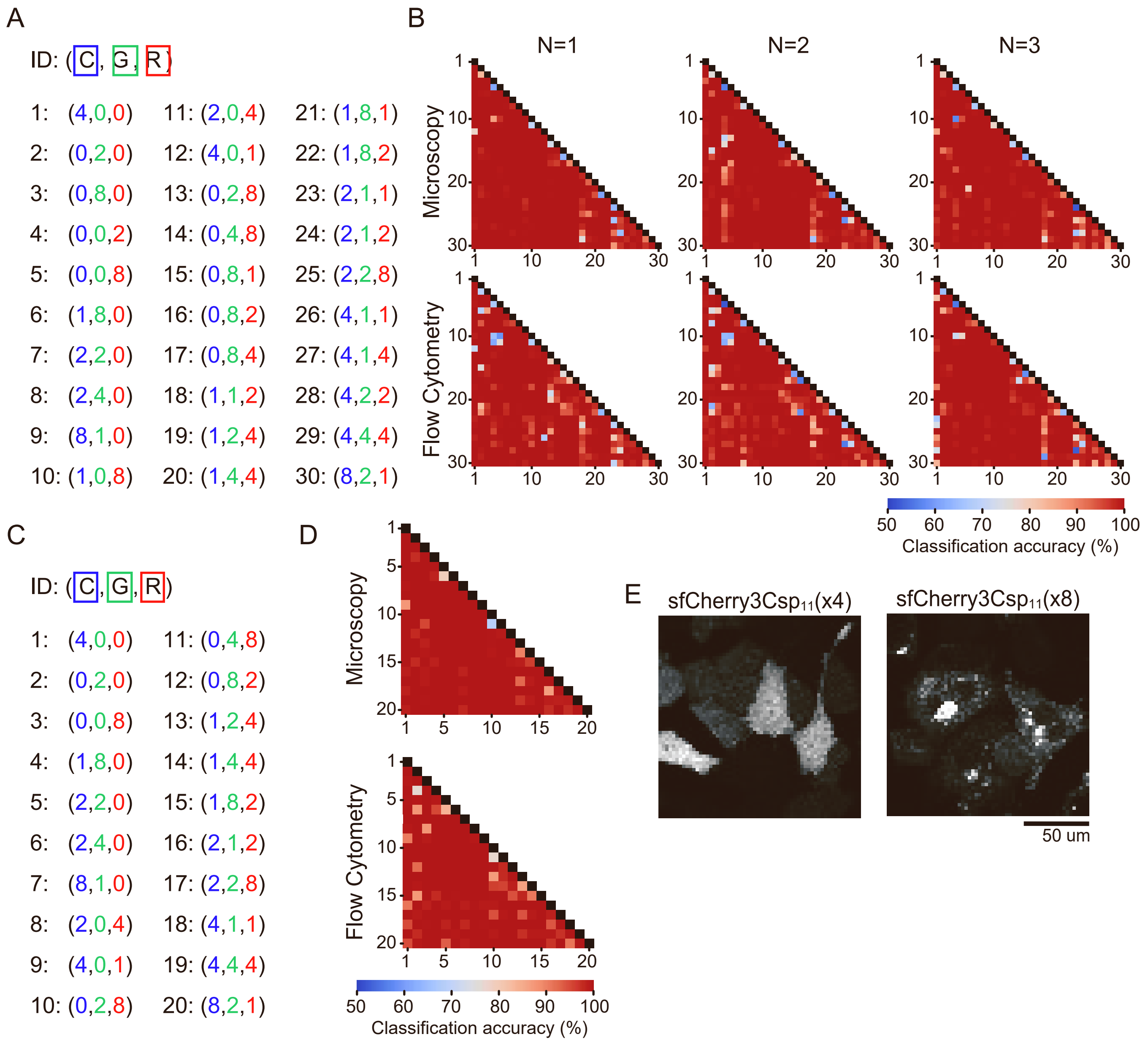

Figure S3: Selection of 20 optimal FP$_{11}$ tags, related to Figure 5.

(A) Catalog of 30 selected FP$_{11}$ tag variants (detailed in Figure 5A), detailing identification numbers and copy numbers of CFP2$_{11}$, mNG3Asp$_{11}$, and sfCherry3Csp$_{11}$ fragments.
(B) Matrix analysis of pairwise classification accuracies for 30 cell populations expressing distinct FP$_{11}$ tag combinations (listed in panel A), evaluated by both microscopy (upper panel) and flow cytometry (lower panel). Data represent results from three independent experiments.
(C) Catalog of optimized FP$_{11}$ tag set comprising 20 selected variants (detailed in Figure 5A), detailing identification numbers and copy numbers of CFP2$_{11}$, mNG3Asp$_{11}$, and sfCherry3Csp$_{11}$ fragments.
(D) Matrix analysis of pairwise classification accuracy for 20 cell populations expressing distinct FP$_{11}$ tag combinations (defined in panel C). Comparative evaluation performed using both microscopy (upper panel) and flow cytometry (lower panel). Data represent mean values from three independent experiments in panel B.
(E) Representative images of sfCherry3Csp fluorescence in HeLa cells expressing ΔSUMOstar-tagged sfCherry3Csp$_{11}$ variants [(x4), or (x8)], with concurrent FP$_{1\text{-}10}$x3 expression.

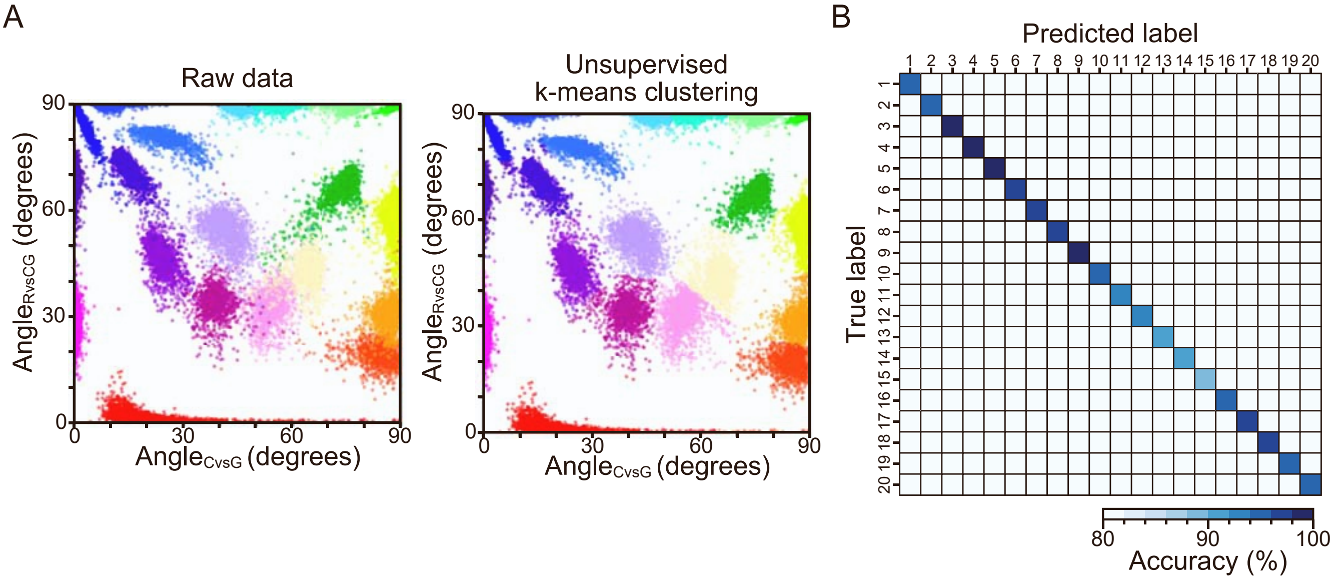

Figure S4: Unsupervised k-means clustering of 20 cell populations, related to Figure 6.

(A) Left: Scatter plot showing the distribution of Angle$_{CvsG}$ versus Angle$_{RvsCG}$ for 20 distinct cell populations (n > 1,000 cells per population). Right: Scatter plot showing the distribution of Angle$_{CvsG}$ versus Angle$_{RvsCG}$ for individual cells grouped into 20 distinct populations using unsupervised k-means clustering.
(B) Classification performance matrix of unsupervised k-means classification in panel A. Color intensity indicates classification accuracy. Overall average accuracy, 96 %.

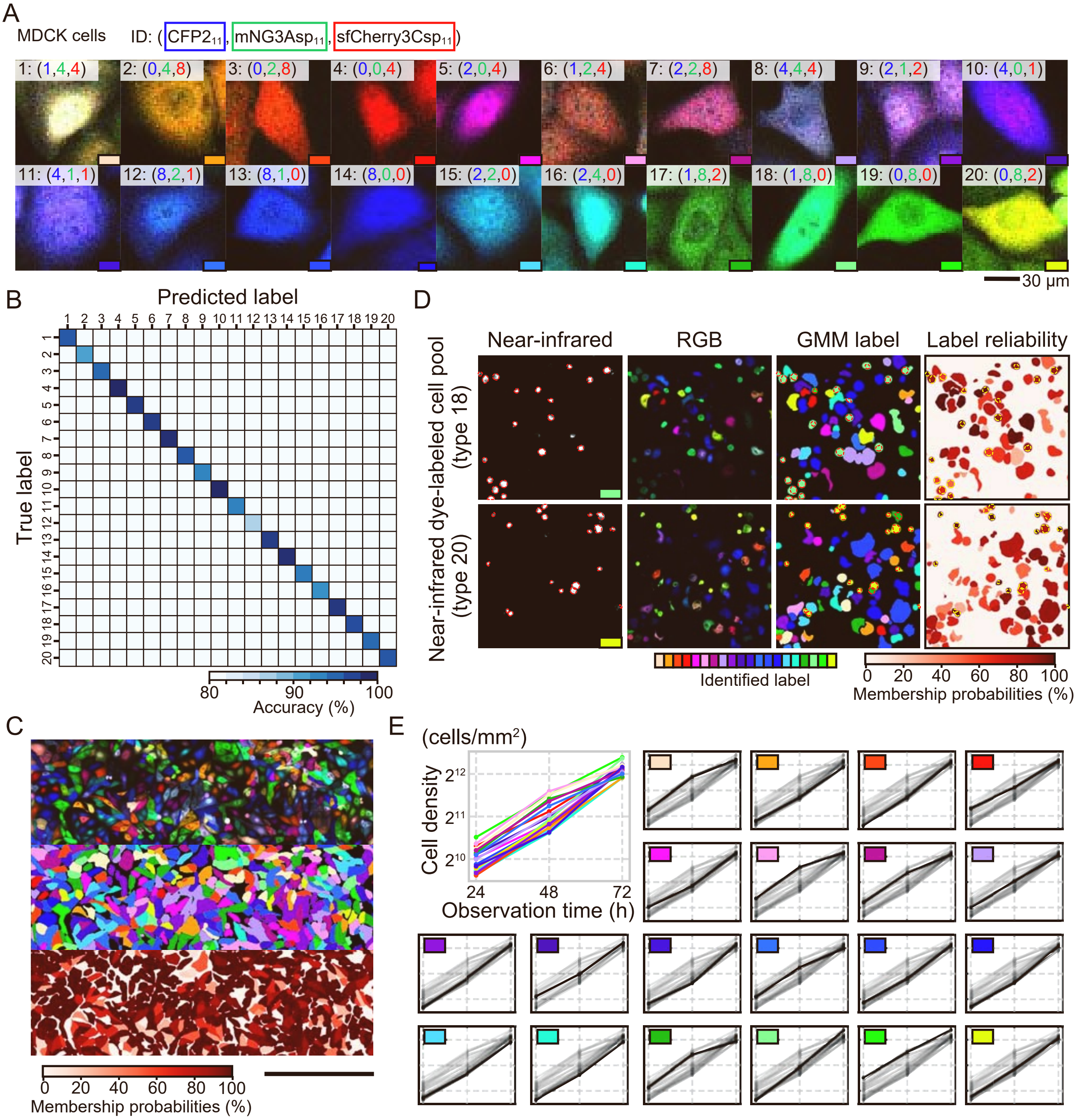

Figure S5: Accurate multicolor classification and normal growth of labeled MDCK cells, related to Figure 7.

(A) Representative multicolor fluorescence micrographs of 20 distinct cell populations expressing FP$_{11}$ tags. Fluorescence channels: CFP2 (blue), mNG3Asp (green), and sfCherry3Csp (red). Copy numbers of each FP$_{11}$ variant (CFP2$_{11}$, mNG3Asp$_{11}$, and sfCherry3Csp$_{11}$) are indicated in the top of each panel. Scale bars: 30 μm.
(B) Classification performance matrix showing prediction accuracy against true population identities (rows). Color intensity indicates classification accuracy. Overall average accuracy, 96%.
(C) Top: Large-field composite image of pooled cell populations from panel (A), displaying CFP2 (blue), mNG3Asp (green), and sfCherry3Csp (red) fluorescence channels. Scale bar: 500 μm. Middle: Population assignment map following GMM-based classification into 20 distinct populations. Bottom: Visualization of GMM classification confidence through membership probability mapping.
(D) From left to right: near-infrared dye–labeled cells stained with DRAQ5, RGB composite image of the pooled 20 cell populations, GMM label map, and GMM label reliability map. The upper panel shows type 18 cells (B,G,R = 1,8,0) and the lower panel shows type 20 cells (B,G,R = 0,8,2), both labeled with the near-infrared dye. Among 81 total cells (type 18, upper panel), the classifier identified 20 true positives, 1 false positive, 1 false negative, and 59 true negatives, yielding a sensitivity of 95.2% (20/21) and specificity of 98.3% (59/60). Among 93 total cells (type 20, lower panel), there were 17 true positives, 0 false positives, 2 false negative, and 75 true negatives, yielding a sensitivity of 89.5% (17/19) and specificity of 100% (75/75).
(E) Growth curves showing cell density of 20 different MDCK cell types fluorescently labeled with the Caterpie method measured every 24 hours. The each panel displays the individual growth curve.

Supplementary Table 1: ***Amino acid sequences of the $FP_{1\text{-}10}$ and FP_{11} tag, related to Figure 6.***

	**Amino acid sequences**
$CFP2_{1\text{-}10}$	MSKGEELFTGVVPILVELEGEVNGHKFSVRGEGEGDATIGKLTLKFICT TGKLPVPWPTLVTTLTWGVQCFSRYPDHMKRHDFFKSAMPEGYVQE RTISFKDDGKYKTRAVVKFEGDTLVNRIVLKGTDFKEDGNILGHKLEYN FNSDNVYITADKQKNGIKANFTIRHNVEDGSVQLADHYQQNTPIGDGP VLLPDNHYLSTQSVLSKDPNEK
$mNeonGreen3Asp_{1\text{-}10}$	MVSKGEEDNMASLPATHELHIFGSINGVDFDMVGQGTGNPNDGYEEL NLKSTKGDLQFSPWILVPHIGYGFHQYLPYPDGMSPFQAAMVDGSGY QVHRTMQFEDGASLTVNYRYTYEGSHIKGEAQVMGTGFPADGPVMT NTLTAADLCVSKMTYPNDKTIISTFKWSYTTVNGKRYRSTARTTYTFAK PMAAKYLKNQPMYVLRKTELKHSM
$sfCherry3Csp_{1\text{-}10}$	MEEDNMAIIKEFMRFKVHMEGSVNGHEFEIEGEGEGHPYEGTQTARL VVTKGDPLPFAWDILSPQFMYGSKAYVKHPADIPDYLKLSFPEGFTWE RVMNFEDGGVVYVTQDSSLQDGQFIYKVKLLGINFPSDGPVMQKKTM GWEASTERMYPEDGALKGEINQRLKLKDGGHYDAEVKTTYRAKKPVQ LPGPYDVDIKLDITSHNED
ΔSUMOstar	MTHINLKVSDGSSEIFFKIKKTTPLRRLMEAFAKRQGKEMDSLTFLYDGI EIQADQTPEDLDMEDNDIIEAHREQ
$CFP2_{11}$	RDHMVLHEYVNAAGIT
$mNeonGreen3Asp_{11}$	TELNFKEWQKAFTDD
$sfCherry3Csp_{11}$	TTIVEQYERAEARHTT
